# An Intracellular Model of Hepatitis B Viral Infection: An In Silico Platform for Comparing Therapeutic Strategies

**DOI:** 10.3390/v13010011

**Published:** 2020-12-23

**Authors:** Farzad Fatehi, Richard J. Bingham, Eric C. Dykeman, Nikesh Patel, Peter G. Stockley, Reidun Twarock

**Affiliations:** 1 York Cross-Disciplinary Centre for Systems Analysis, University of York, York YO10 5GE, UK; farzad.fatehichenar@york.ac.uk (F.F.); r.j.bingham@york.ac.uk (R.J.B.); eric.dykeman@york.ac.uk (E.C.D.); 2 Department of Mathematics, University of York, York YO10 5DD, UK; 3 Department of Biology, University of York, York YO10 5NG, UK; 4 Astbury Centre for Structural Molecular Biology, University of Leeds, Leeds LS2 9JT UK; fbsnpat@leeds.ac.uk

**Keywords:** hepatitis B virus, intracellular model, antiviral therapy, packaging signal, virus assembly

## Abstract

Hepatitis B virus (HBV) is a major focus of antiviral research worldwide. The International Coalition to Eliminate HBV, together with the World Health Organisation (WHO), have prioritised the search for a cure, with the goal of eliminating deaths from viral hepatitis by 2030. We present here a comprehensive model of intracellular HBV infection dynamics that includes all molecular processes currently targeted by drugs and agrees well with the observed outcomes of several clinical studies. The model reveals previously unsuspected kinetic behaviour in the formation of sub-viral particles, which could lead to a better understanding of the immune responses to infection. It also enables rapid comparative assessment of the impact of different treatment options and their potential synergies as combination therapies. A comparison of available and currently developed treatment options reveals that combinations of multiple capsid assembly inhibitors perform best.

## 1. Introduction

The hepatitis B virus is a major global health burden. According to the Hepatitis B Foundation, ∼2 billion people have been infected with the hepatitis B virus (HBV), resulting in ∼292 million chronic infections that lead to ∼884,000 deaths each year from virally related complications, such as liver cancer and cirrhosis. Despite the availability of an excellent prophylactic recombinant vaccine, ∼30 million people will become newly infected each year [1]. The vaccine is not curative, and infected individuals are currently treated with drugs targeting the viral polymerase. These come with a risk of eliciting resistance mutations, even, in some cases, within months of starting therapy [2]. Clearing the virus from patients is currently prevented because of the persistence of a covalently closed circular DNA (cccDNA) episome of the viral genome, which acts as a source of novel virus formation. Achieving the goal of eliminating viral hepatitis as a major public health threat by 2030 will require a systematic evaluation of existing therapeutic options and novel forms of antiviral therapy, both current and yet to be discovered, including the potential synergies of combination therapies [3].

The in silico approach presented here is designed to support these efforts. Based on the current understanding of the HBV intracellular lifecycle, we developed a mathematical model grounded in experiments that permits rapid and cheap evaluation of antiviral drug strategies. This is particularly important because success in meeting the global challenge may require targeting multiple aspects of the viral lifecycle with potentially unexpected synergistic effects. Whilst some aspects of intracellular HBV dynamics have been modelled previously [4,5,6], our model is the first that takes into account reactions describing all the processes targeted by different types of antiviral strategies [3,7], including details of the assembly of both infectious and non-infectious particles and the mechanism of nucleocapsid assembly [8]. In particular, the model includes the formation of the sub-viral particles (SVPs) that vastly outnumber infectious virions. The model is based on a Gillespie algorithm and reaction networks. It can therefore capture the kinetics of particle formation at the low molecular species concentrations typical at the start of an infection. This reveals unsuspected features in SVP dynamics that could play important roles for immune recognition [9,10]. We also demonstrate that the model correctly captures experimental/clinical outcomes in a comparative analysis of licensed HBV drugs, and provides an assessment for drug options that are currently in the developmental phase. Among others, our results reveal significant synergistic effects between capsid assembly modulators (CAMs) that are already being developed and gRNA packaging signal (PS)-targeting drugs that we are developing, suggesting novel forms of combination therapy.

## 2. Materials and Methods

### 2.1. An In Silico Model of Intracellular HBV Infection Dynamics

**Figure 1:**
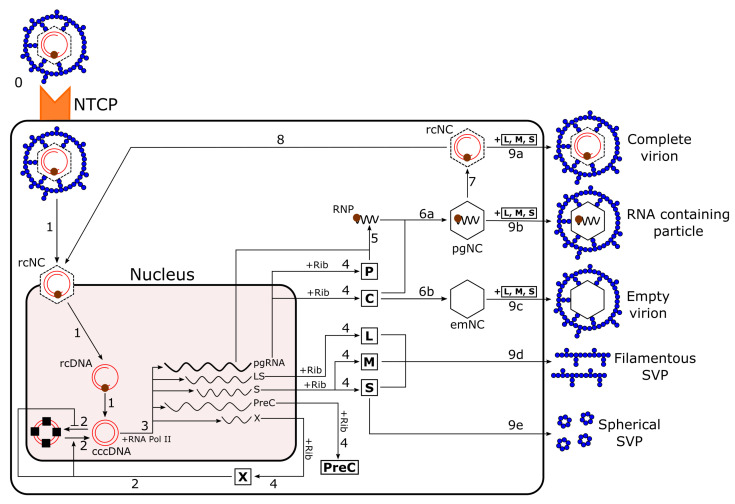
The hepatitis B virus (HBV) life cycle.Step 0: Viral entry is mediated by the sodium-taurocholate co-transporting polypeptide (NTCP) receptor, which allows the nucleocapsid (rcNC) containing relaxed circular DNA (rcDNA) to be released into the cell via endocytosis. This step is not included explicitly in the model. Step 1: After attachment of the rcNC to the nucleus, it delivers the rcDNA. The host DNA repair factors convert the rcDNA into covalently closed circular DNA (cccDNA). Step 2: X protein, which gets produced by the cccDNA over the course of infection, promotes the de-silencing of the cccDNA and blocks its silencing. Step 3: The cccDNA is used as a template by RNA polymerase II (RNA Pol II) to synthesise viral RNAs, including the pgRNA; LS, S, PreC, and X mRNAs. The pgRNA encodes both the core (C) and polymerase (P) proteins; X mRNA the X protein; LS mRNA the L surface protein; S mRNA the M and S surface proteins; and PreC mRNA the precore (PreC) protein. Step 4: Translation of these mRNAs by ribosomes (Rib) leads to synthesis of viral proteins. Step 5: The pgRNA and P form a 1:1 ribonucleoprotein (RNP) complex, which is assembly competent. Step 6a: Encapsidation of the RNP complex by C proteins to form a nucleocapsid containing pgRNA:P (pgNC), also termed the immature nucleocapsid. Step 6b: Assembly of C proteins into an empty nucleocapsid (emNC). Step 7: The reverse transcription of pgRNA by P within the pgNC resulting in the conversion of pgNC into an rcDNA-containing nucleocapsid (rcNC), which is also termed the mature nucleocapsid. Step 8: Recycling of the rcDNA from mature nucleocapsids to form more cccDNAs. Step 9a: Envelopment of mature nucleocapsids within a membrane layer containing the surface proteins L, M, and S, leading to the secretion of a complete virion. Step 9b: Envelopment of an immature nucleocapsid resulting in secretion of an RNA-containing particle. Step 9c: Envelopment of an empty nucleocapsid and secretion of an empty virion. Step 9d: L, M, and S proteins form empty filaments and filamentous subviral particles (SVPs) via conventional tubular budding into late endosomes and exit the cell. Step 9e: S proteins assemble into octahedral spheres (spherical SVP) and exit the cell.

We have developed a comprehensive stochastic model for intracellular dynamics of HBV infection. The model consists of essential steps of the viral life cycle, which are shown in Figure 1. Similarly to intracellular models of hepatitis C virus [11], in this model, the viral entry step (Step 0; Figure 1), in which the virus releases its nucleocapsid (rcNC) into the cell through the sodium taurocholate cotransporting polypeptide (NTCP) receptor [12], is not included explicitly, as our focus is on the intracellular infection dynamics. We use our model to study the virion release kinetics from a single infected cell in unprecedented detail, and to present a model in which to test antiviral strategies and explore their synergies. We have used discrete reaction networks to develop our model, rather than continuous equations, enabling detailed study of the onset of the infection when particle numbers are low. The model and its advantages over previous models are described briefly in the following. A comprehensive account of the model, including all reactions and parameter choices, is provided in the Supplementary Material.

*cccDNA production*: The first step after viral entry is the formation of cccDNA (Step 1; Figure 1). Murray and Goyal [6] have studied cccDNA formation in their model, which includes delivery of cccDNA by the nucleocapsid to the nucleus. In their model, a stochastic agent-based approach using anordinary differential equation (ODE), this step is represented as a single reaction, describing the net result of three discrete reactions (Step 1; Figure 1) that we are modelling here. Firstly, there is the intracellular trafficking where the rcNC moves toward the nucleus. Secondly, the rcNC releases its cargo, which is a relaxed circular DNA (rcDNA), into the nucleus [13]. We assume that these two reactions occur as one reaction with rate α. Then, the rcDNA is converted into cccDNA (at rate *a*). This conversion occurs via cellular enzymes that are normally involved in base excision repair of DNA damage [9,14] akin to correction of “damaged” DNA. Splitting this step into two reactions is important because capsid assembly modulators (CAMs) can bind to the rcNC and disassemble it [15]. In addition, using anti-host DNA repair factors, which is presented as a possible future treatment [7], will suppress the last reaction of this step. Therefore, we used two reactions for these steps, enabling us to study the impact of the two treatment options, and present a comparison between them.

*Epigenetics of cccDNA*: cccDNA is organised into mini-chromosomes with host cell histones and potentially other host and viral proteins [16,17]. Therefore, transcription from cccDNA is subject to epigenetic regulation, providing numerous options for dynamic epigenetic control of cccDNA transcriptional activity [18,19,20]. In the absence of the X protein (HBx), which gets produced by the cccDNA over the course of the infection, cccDNA appears to be rapidly silenced [21]. In addition, HBx promotes the de-silencing of cccDNA [7] (Step 2; Figure 1). We model (de-)silencing of cccDNA with rate ($\lambda_{off}/\left(1+s\bar{X}\right)$) $\lambda_{on}\left(1+s\bar{X}\right)$, where *s* indicates the efficiency of the available numbers of HBx proteins ($\bar{X}$) [22]. This step is not included in previous models. However, targeting the X protein to keep the cccDNA silenced is suggested as a possible therapeutic strategy [7], and it is therefore included in our model.

*Transcription of cccDNA*: cccDNA serves as a template for transcription via host RNA polymerase II (Step 3; Figure 1), producing: pregenomic RNA (pgRNA; also called the C (core) mRNA [23]) coding for core (C) and polymerase (P) proteins; X mRNA coding for the X protein; LS mRNA coding for the L surface protein; S mRNA coding for the M and S surface proteins; and PreC mRNA coding for the precore protein, which forms E protein (HBeAg). Clinically, measuring the level of HBeAg can be used as an indicator for the phases of chronic infection, which could be included in a within-host model [24]. However, as we are focusing on the formation of new particles over the infection of a single cell and HBeAg is a non-structural protein, it is not included in the simulation results. Nakabayashi [4,5] includes this step in his model, covering only pgRNA and S mRNA production from cccDNA. However, the inclusion of all viral mRNA species in the model is important due to the roles of their products in the viral life cycle, such as X protein in the activation of cccDNA, or the surface proteins in the envelopment of nucleocapsids. We therefore model transcription by two sets of five reactions, describing complex formation between cccDNA and RNA polymerase II, followed by mRNA production and cccDNA release from the complex (Supplementary Equation (S1)) [25]. In this way, we can capture the delay of this process for the production of each RNA, and we can study the impact of the number of RNA polymerase II (RNA Pol II) molecules on virus dynamics. RNA Pol II and cccDNA bind together at rate b to produce pgRNA. Since the level of surface protein is higher than the HBV DNA level, we assume the binding rates that lead to the production of the LS and S mRNAs to be $n_{Ld}$ times larger [6].

*Viral protein synthesis*: After export from the nucleus to the cytoplasm, the viral mRNAs are translated (Step 4; Figure 1) by ribosomes. This is modelled by analogy to the transcription step (Supplementary Equation (S2)) and has similar advantages [25]. Ribosome and pgRNA bind at rate *d* to produce polymerase (P) protein.

*rcDNA-containing nucleocapsid formation*: This process contains three steps: Firstly, P protein and pgRNA form a ribonucleoprotein (RNP) complex (Step 5; Figure 1) [13,23,26]. This is mediated by a short structured RNA signal located at the $5^{\prime }$ end of the pgRNA, called ε, that is recognised specifically by P [27,28,29,30]. Secondly, nucleocapsid assembly from core (C) protein dimer occurs around the RNP complex (Step 6a; Figure 1) [8]. Then, the reverse transcription step occurs (Step 7; Figure 1), resulting in the formation of an rcDNA-containing nucleocapsid (mature nucleocapsid; rcNC) [23]. For the formation of rcNCs, Nakabayashi [4,5] considered just two of these reactions, corresponding to the formation of RNP from P and pgRNA and of rcNC from core protein and RNP. Murray and Goyal [6] only considered one of these reactions, the production of rcNCs from cccDNA, as a linear function. However, the formation of a nucleocapsid around the RNP complex is promoted by at least three packaging signals (PSs; denoted as PS1, PS2, and PS3) in the pgRNA, which bind to the core protein (CP) dimer [8], as illustrated in Figure 3a. A number of small-molecular-weight compounds have been identified that can bind to these three PSs, inhibiting nucleocapsid assembly in vitro. In addition, CAMs can bind to C proteins and thus alter the kinetics and pathway of C protein assembly [15]. In order to be able to study the impact of these nucleocapsid assembly inhibitors and compare their action, we included unprecedented details of the assembly process in our model. Moreover, in addition to complete virions (infectious particles), an infected cell produces incomplete particles (fully formed particles without a DNA genome). Incomplete particles occur in three distinct forms: RNA-containing particles; empty virions; and subviral particles (SVPs) [23]. SVPs assemble from surface proteins (L, M, and S) only, and are either filamentous or spherical with diameters of about 20 nm [31,32]. Incomplete particles deplete the resources and can act as decoys for the immune response [10]. Therefore, modelling their assembly and revealing their release kinetics are important. The modelling of these steps is described below:

We model the formation of the RNP complex, which is the basis for nucleocapsid assembly, with rate *g*. The assembly step (Step 6a; Figure 1) is promoted by three PSs. We assume that CP dimer binding to PSs contributes to the formation of a nucleation complex; Figure 3b shows all possible pathways for this, where R denotes the RNP complex and C${}_{j}$ (j=1,2,3) indicate PS*j* bound to a CP dimer. Reactions between complexes in which different combinations of these PSs are bound are shown as black arrows, representing the binding/unbinding of CP (Figure 3b). Once all PSs are bound to CP, that is, when the RC${}_{1}$C${}_{2}$C${}_{3}$ complex is formed, more CP dimers (denoted as C) are recruited to build the fully formed pgRNA-containing nucleocapsid (pgNC), which is also called the immature nucleocapsid. In our model, we assume that nucleocapsids are made of 120 C proteins following a T=4 architecture, ignoring the T=3 particles that occur in only 5% of cases [8]. Thus, we consider 117 reactions, describing the binding of individual C proteins to the RC${}_{1}$C${}_{2}$C${}_{3}$ complex to create the pgNC. At this stage, the pgRNA serves as a template for the reverse transcription reaction, leading to the formation of rcDNA with rate $s_{1}$ (Step 7; Figure 1), and ultimately to assembly of the rcDNA-containing mature nucleocapsid (rcNC).

In ∼10% of immature nucleocapsids, the reverse transcription leads to the synthesis of a double-stranded linear DNA (dslDNA) that can be integrated into the host cell genome, where it acts as a template for LS mRNA, S mRNA, and X mRNA. We note that dslDNA can also be circularised via the non-homologous end-joining (NHEJ) DNA repair pathway [33,34]. However, we do not integrate dslDNAs into the model and only consider rcDNAs because the production rate of dslDNA is much lower than that of rcDNA. Integration of dslDNA occurs approximately once per ∼$10^{5}-10^{6}$ infected cells [35], and many cccDNAs produced from dslDNA are functionally defective, as the NHEJ pathway is error-prone [7].

*Envelopment and secretion of complete and incomplete particles*: At low L protein levels, the rcNC can deliver its contents to the nucleus to amplify the cccDNA reservoir (Step 8; Figure 1) [6]. By contrast, when the level of L protein is sufficiently high, rcNCs acquire the viral surface proteins (L, M, and S) and exit the cell via the cellular secretory pathway (Step 9a; Figure 1) [13,36]. In order to reflect this dependence on the level of L protein, the delivery of the rcNCs’ content to the nucleus is modelled at rate $te^{-\lambda L}$ (Supplementary Equation (S3)), whilst the rcNC release rate is $t(1-e^{-\lambda L})$ [6,37]. Moreover, since a complete virion collects the surface proteins during budding, we consider its secretion as a single reaction in the model:

(1) $$\mathrm{rcNC}+80S+20M+20L\overset{t(1-e^{-\lambda L})}{\to }\mathrm{complete}\;\mathrm{virion}\;.$$

The protein stoichiometry has been chosen at a relative ratio of 1:1:4 for L, M, and S as in the filamentous SVPs [38], as they are secreted from the same compartment [39]. Given that about 210 to 270 surface protein subunits have been reported per viral particle [31,40], and that both core and surface proteins occur as dimers [41], we assume a 1:1 stoichiometry between the core and the surface proteins, resulting in the ratio 20:20:80 for L, M, and S.

Secretion of RNA-containing particles occurs at low levels. These particles are immature nucleocapsids that have not undergone the reverse transcription step, but still acquire the viral surface proteins and exit the cell [3,23,42,43,44,45] (Step 9b; Figure 1). Moreover, 120 C proteins can assemble into empty nucleocapsids (Step 6b; Figure 1) [46,47] and exit the cell as empty virions. Filamentous SVPs, also called L-rich subvirals [41], are formed via conventional tubular budding [39,48] upon release. The secretion of all these particles is modelled akin to that of complete virions (Supplementary Equations (S4)–(S6)). However, secretion of spherical SVPs (octahedral particles made of 48 S proteins) has a different pathway: S proteins attached to the membrane of the endoplasmic reticulum (ER) self-assemble into filaments with diameters of about 20 nm [41]. These filaments are transported to the roughER–Golgi intermediate compartment (ERGIC), where they convert into spheres before exiting the cell (Step 9e; Figure 1) [32,41,49]. In the model, we assume that as soon as 48 S proteins are assembled, they are released as a spherical particle at rate $t_{2}$. It has also been reported that the production and release of spherical versus filamentous particles depends on the concentration of L protein, with lower values favouring spherical SVPs [37,39,50]; this impact of the L protein is captured in the initiation reaction (Supplementary Equation (S7)).

### 2.2. Modelling of Different Drug Treatments

**Figure 2:**
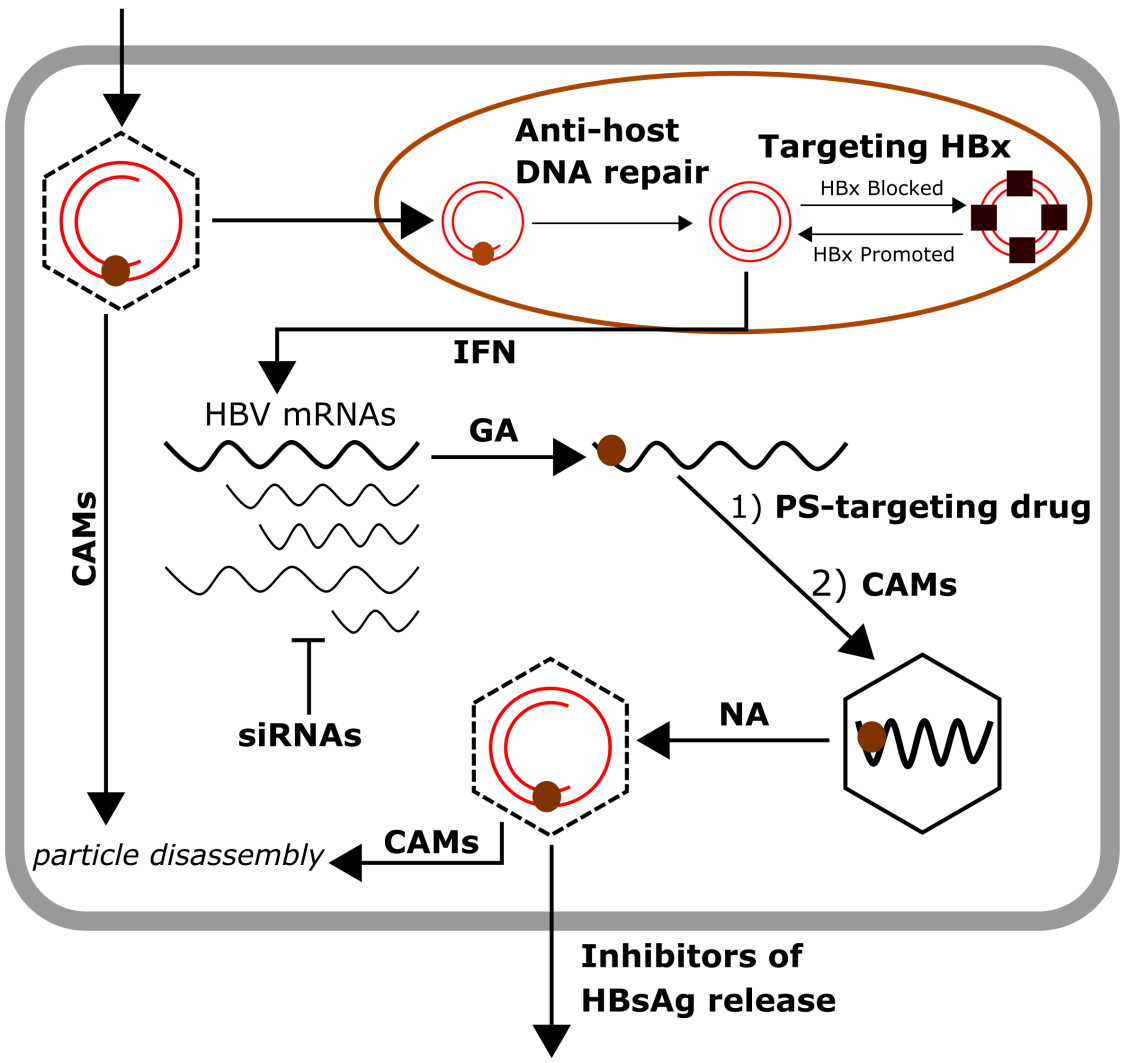
Current and potential future drugs for the treatment of HBV. The different types of drugs are labelled next to the viral life cycle steps they target. Capsid assembly modulators (CAMs) both inhibit nucleocapsid assembly and promote disassembly of mature nucleocapsids, either following entry or prior to release, and are therefore indicated at multiple steps of the viral life cycle. Interferon (IFN)-based therapy blocks the transcription step. Small interfering RNAs (siRNAs), which reduce mRNA levels, are indicated by the bar-headed line. Geldanamycin (GA) prevents the formation of the ribonucleoprotein (RNP) complex. Nucleot(s)ide analogues (NAs) inhibit the progression of immature into mature nucleocapsids. Packaging signal (PS)-targeting drugs bind to the PSs of HBV and inhibit nucleocapsid assembly. Inhibitors of HBsAg release target viral and subviral particle (SVP) egress.

Figure 2 shows all currently available and possible future treatment options for chronic hepatitis B [3,7]. Since our comprehensive model covers all reactions that can be targeted by these drugs, we can study their effects and explore synergies. The impact of these drugs is modelled as follows.

Interferon (IFN)-based therapy comprises stand-alone IFN-α orpegylated interferon-α (Peg-IFN-α) [51]. IFN-α results in cccDNA-bound histone hypoacetylation as well as active recruitment of transcriptional corepressors to the cccDNA and reduces binding of transcription factors to an active cccDNA [19]. It also degrades cccDNA via the activation of APOBEC3A in infected cells [52,53,54]. Thus, we model the action of IFN-α binding as silencing of the cccDNA:

$$\begin{array}{cc} \; & \mathrm{IFN}+\mathrm{cccDNA}\overset{\;I_{bind}}{\underset{\;I_{unbind}}{⇄}}\mathrm{ccc}:\mathrm{IFN}. \end{array}$$

In addition, the presence of IFN-α increases the cccDNA degradation rate:

$$\begin{array}{c} \mathrm{cccDNA}\overset{\mu (1+\psi \mathrm{IFN})}{\to }0. \end{array}$$

Here, IFN denotes the amount of IFN-α, ψ is the efficacy of this drug in cccDNA degradation, and $I_{bind}$ and $I_{unbind}$ are the binding and unbinding rates of IFN-α, respectively. Note that IFN-α also acts via the activation of natural killer (NK)/NKT cells, but these effects must be studied in an intercellular model. However, this treatment option is associated with significant side effects [55,56].

Geldanamycin (GA) prevents the formation of the RNP complex [26], mediated by its binding to heat shock protein 90 (Hsp90) [57,58,59], which is required for association of P with the pgRNA. Assuming that Hsp90 is not rate limiting, we model the formation of a P:GA complex from P and GA that cannot bind to the pgRNA:

$$P+GA\overset{k_{bind}}{\underset{k_{unbind}}{⇄}}P:GA,$$

where $k_{bind}$ and $k_{unbind}$ indicate the binding and unbinding rates of GA.

Nucleot(s)ide analogues (NAs), such as lamivudine, adefovir, entecavir, tenofovir, telbivudine, famciclovir, and clevudine [51], incorporate into growing DNA strands and thus inhibit the progression from immature to mature nucleocapsids [3,7]. This is modelled as NAs binding to the pgNC, stopping the process of reverse transcription:

$$\begin{array}{cc} \; & \mathrm{pgNC}+\mathrm{NA}\overset{l_{bind}}{\underset{l_{unbind}}{⇄}}\mathrm{pgNC}:\mathrm{NA},\\ \; & \mathrm{pgNC}:\mathrm{NA}+80S+20M+20L\overset{t_{1}\left(1-e^{-\lambda L}\right)}{\to }\mathrm{RNA}-\mathrm{containing}\;\mathrm{particle}, \end{array}$$

where $l_{bind}$ and $l_{unbind}$ are the binding and unbinding rates of NAs to pgNC.

A further class of drugs is that of virus assembly inhibitors, which target core protein assembly. Capsid assembly modulators (CAMs), also called core protein allosteric modulators (CpAMs), bind to C protein and thus alter the kinetics and pathway of C protein assembly [15,60]. This either leads to the formation of morphologically intact empty nucleocapsids (class I mechanism of action (MoA) compounds), or to aberrant nucleocapsids devoid of pgRNA (class II MoA compounds) [61]. These drugs can also inhibit the formation of cccDNA due to an accelerated breakdown of nucleocapsids in the cytoplasm [62,63]. It has been shown that elongation of positive-stranded DNA induces structural changes in the nucleocapsid that cause mature nucleocapsids to bind CAMs, triggering their disassembly [15]. Moreover, CAMs efficiently inhibit the replication of HBV mutants resistant to NAs [64,65] and are active against multiple HBV genotypes [61]. Several CAMs are in preclinical evaluations or have entered clinical trials. Lahlali et al. [63] have studied the impact of the novel CAMs JNJ-827 and JNJ-890, which are class I and II, respectively, and are potent inhibitors of HBV replication with respective half-maximal effective concentrations of 4.7 and 66 nM, respectively.

We model CAM (un)binding to core protein C as:

$$C+\mathrm{CAM}\overset{C_{bind}}{\underset{C_{unbind}}{⇄}}C:\mathrm{CAM},$$

where ($C_{unbind}$)$C_{bind}$ are the (un)binding rates. Moreover, C:CAM complexes can associate to form empty nucleocapsids. As JNJ-827, which is class I, has a much lowerEC50 (the concentration of a drug that gives half-maximal response), we only model the class I scenario. C:CAM complexes form morphologically intact nucleocapsids requiring 120 units. We model the formation and secretion of empty nucleocapsids as:

$$\begin{array}{cc} \; & C:\mathrm{CAM}+jC:\mathrm{CAM}\overset{\kappa_{nc}}{\to }(j+1)C:\mathrm{CAM},\;j=1,\ldots ,\left(nuc-1\right),\\ \; & C:\mathrm{CAM}+jC:\mathrm{CAM}\overset{\kappa_{1}}{\to }(j+1)C:\mathrm{CAM},\;j=nuc,\ldots ,118,\\ \; & 119C:\mathrm{CAM}+C:\mathrm{CAM}\overset{\kappa_{1}}{\to }\mathrm{emNC}:\mathrm{CAM},\\ \; & \mathrm{emNC}:\mathrm{CAM}+80S+20M+20L\overset{t(1-e^{-\lambda L})}{\to }\mathrm{empty}\;\mathrm{virion}, \end{array}$$

where nuc is the number of core proteins in the nucleation complex [46,66], and $k_{nc}$ and $\kappa_{1}$ are the forward rates for the nucleation and elongation reactions, respectively.

As suggested in [15], CAMs can also bind to rcNCs and trigger their disassembly, followed by degradation of the rcDNA. We assume that dissociated nucleocapsids are partially assembled; therefore, C protein in these intermediates is not available for formation of additional nucleocapsids. Thus:

$$\begin{array}{cc} \; & \mathrm{rcNC}+\mathrm{CAM}\overset{C_{bind}}{\underset{C_{unbind}}{⇄}}\mathrm{rcNC}:\mathrm{CAM},\\ \; & \mathrm{rcNC}:\mathrm{CAM}\overset{k_{dis}}{\to }\mathrm{CAM}, \end{array}$$

where $k_{dis}$ indicates the disassembly rate.

In order to target the role of PSs in nucleocapsid formation, a number of small-molecular-weight compounds have been identified that can bind to the three packaging signals of HBV and inhibit nucleocapsid assembly in vitro. The impact of these novel nucleocapsid assembly inhibitors is modelled via the (un)binding to PSs using experimentally measured affinities (Supplementary Table S2), as described in detail in the Supplementary Material.

Novel therapeutic strategies: Novel, potentially curative therapies for HBV infection are either evaluated in preclinical models or are in the first phases of clinical development [3,7]. These include entry inhibitors that reduce the infection rate of target cells; anti-host DNA repair factors that inhibit the conversion of rcDNA into cccDNA by targeting the DNA repair factors; cccDNA and HBx protein targeting drugs that increase or decrease the silencing and de-silencing rates of cccDNA, respectively; small interfering RNAs (siRNAs) that create an RNA-induced silencing complex (RISC) with mRNAs and cleave them; and inhibitors of viral and SVP release that target the viral and SVP egress. Our detailed model of the HBV life cycle contains all reactions pertinent to the processes targeted by these drugs, enabling a comparative analysis of these possible future treatment options with the already available drugs.

## 3. Results

### 3.1. Novel Insights into Infection Kinetics in the Drug-Free Situation

**Figure 3:**
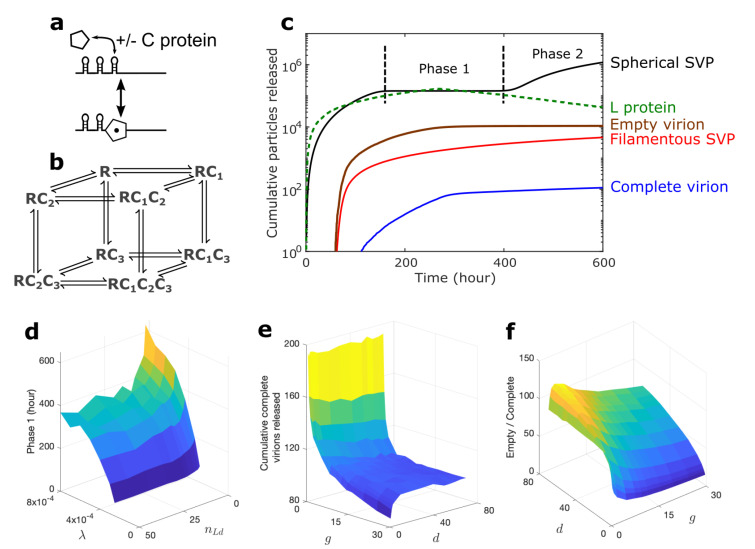
The release kinetics indicate a novel two-phase behaviour in the drug-free case. (**a**) The interaction between PSs and C proteins where C proteins bind and unbind to the RNP (R) complex. (**b**) All possible pathways to occupying all three PSs. (**c**) The time evolution of the model shows the cumulative number of released particles, averaged over 100 simulations. The 95%CI (the confidence interval, shown as shaded area around each curve) is included, but is not visible due to the small size and the logarithmic scale. This can be seen more clearly in Figure 5 and Figure S11. Phase 1 is the plateau in the release of spherical SVPs. The average number of RNA-containing particles released over 100 simulations is 0.1 (0.1±0.07 95% CI), which is not visible here due to the scale, but is shown in Figure S11d. Although L protein does not get released, its concentration is plotted (green dashed line) to show the impact of L protein on the release of particles and the emergence of Phase 1. (**d**) The length of Phase 1 as a function of λ and $n_{Ld}$ (Supplementary Equations (S1) and (S3)). (**e**) The total number of complete virions released from an infected cell over 600 h after cccDNA formation. (**f**) The ratio of the number of empty virions over complete virions released over 600 h after cccDNA formation as a function of *g*, which is the binding rate of P protein to pgRNA, and *d*, which is the binding rate of the ribosome to the pgRNA (Supplementary Equation (S2)). This figure is plotted using parameter values from Supplementary Table S1.

**Figure 4:**
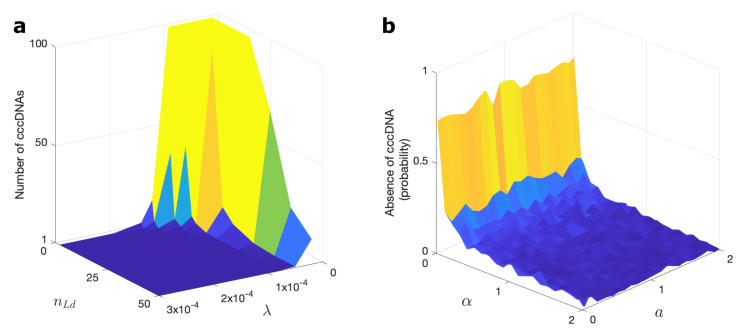
Prediction of cccDNA amplification and revealing rcDNA delivery step as the most crucial reaction in cccDNA formation. (**a**) The average number of cccDNAs in an infected cell as a function of λ and $n_{Ld}$ (Supplementary Equations (S1) and (S3)). The increase in the number of cccDNAs happens due to the intracellular cccDNA amplification step (Step 8; Figure 1). (**b**) The probability of prevention of cccDNA formation over 10,000 simulations after viral entry in a drug-free case. We just consider the cccDNA formation step (Step 1; Figure 1) when one rcNC is entered into a cell. Probability indicates the number of simulations where cccDNA did not get formed over 10,000. This figure is plotted using parameter values from Supplementary Table S1.

The cumulative number of infectious and noninfectious particles released over time was computed as an average over 100 stochastic simulations of the reaction network (Figure 3c) using the Gillespie algorithm [67] implemented in Fortran and parameters adapted from the experimental literature (Supplementary Table S1) [68]. We assume infected cells to have a lifespan of approximately 600 h. This is an implicit way of accounting for the fact that an infected cell is the target of innate and adaptive immune responses that reduce its lifetime below that of the lifespan of the cccDNA of around 1200 h [6]. For parameters not covered in the experimental literature, a sensitivity analysis was performed to ensure that model outcomes are not biased by parameter choices (Supplementary Figures S1–S9). Consistently with experimental results, the most abundant species are the spherical SVPs (Figure 1 [31,49]) that are released prior to other particle types. They act as decoys binding anti-HBV antibodies. Interestingly, their release profile shows a two-phase behaviour that has not been reported before. When the first intact virions are released, after about 90 h post-infection, the release of the SVPs stalls (see the Phase 1 plateau in Figure 3c) until the onset of a second release phase (Phase 2 in Figure 3c). The latter phase coincides with a drop in the concentration of L proteins within the cell (green dashed line in Figure 3c). Additionally, during Phase 1, the level of S proteins in a complex decreases before we observe the release of complete virions, as seen in Supplementary Figure S10. The length of Phase 1 increases with the rate of particle release, λ (Figure 3d). Our sensitivity analysis indicates that this two-phase behaviour is prevalent, except for small (<$2\times 10^{-4}$) values of λ. However, as λ impacts the number of cccDNAs formed (Figure 4a), these small values of λ are associated with higher numbers of cccDNA per cell. cccDNA levels rarely exceed one copy per cell [33], fixing $\lambda >2\times 10^{-4}$. For these values of λ, the model always displays the two-phase behaviour (Figure 3d), suggesting that it is biologically significant. In HBV/HIV coinfection, it has been observed that the number of cccDNAs can sometimes be as high as 10–16 cccDNAs per cell [69] (although the median was still found to be one). However, due to the complexity of these coinfections, this is beyond the scope of this model. We know that empty virions are typically found at 100-fold higher levels than complete virions [23]. Figure 3e indicates that although decreasing *g* increases the total number of released complete virions, the ratio of the number of released empty virions over complete virion falls below 100 (Figure 3f). Increasing *g* or decreasing *d*, have the same effects. Thus, in our simulations, we assume $d=40\;{\mathrm{molecule}}^{-1}h^{-1}$ and $g=5\;{\mathrm{molecule}}^{-1}h^{-1}$, which provides a more realistic viral dynamic.

### 3.2. Modelling Synergies between Antiviral Strategies

**Figure 5:**
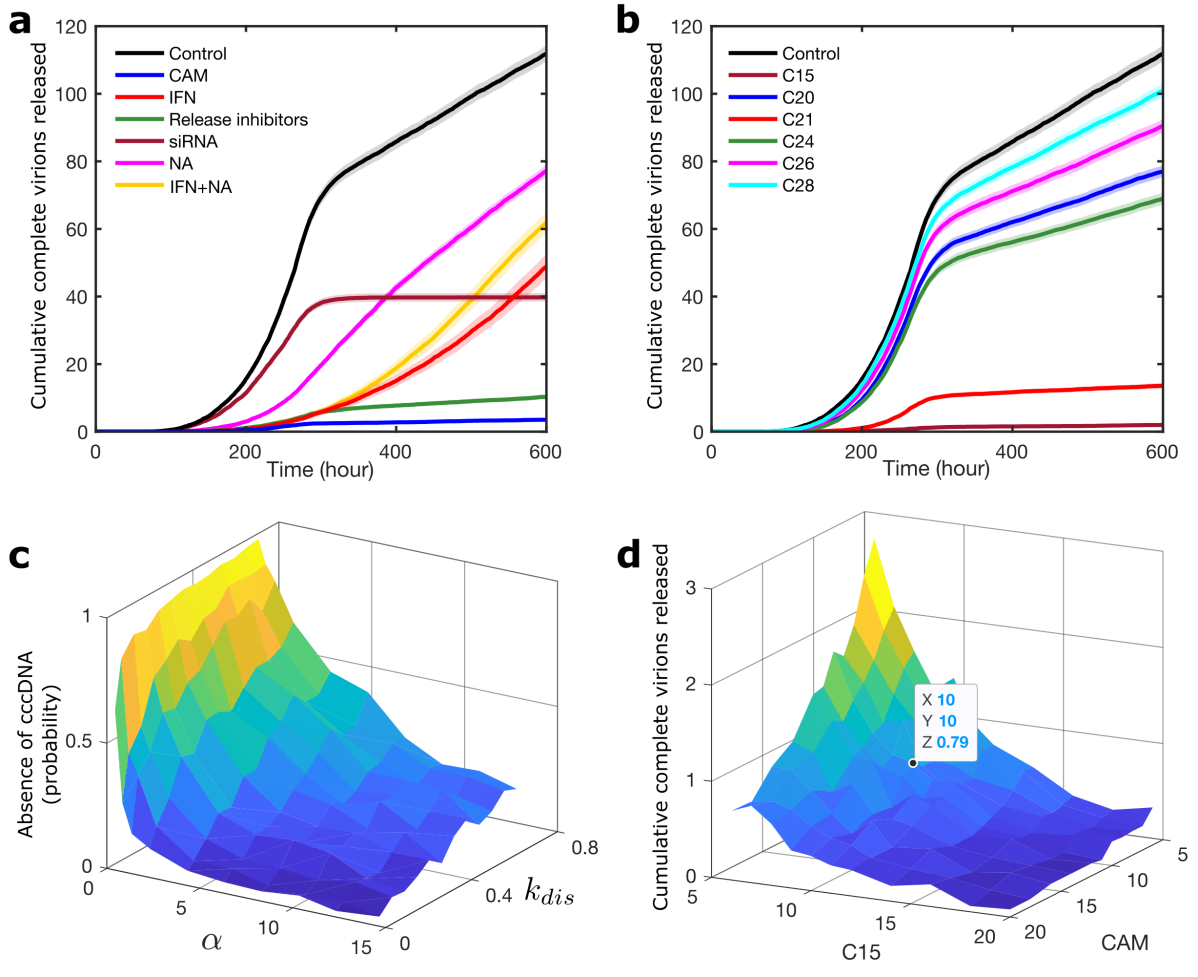
Comparative analysis of all possible treatment options for HBV, revealing a synergistic effect between nucleocapsid assembly inhibitors. (**a**) The dynamics of complete virions released with different treatment options after cccDNA formation for treatment starting once the initial condition cccDNA = 1 is reached in the model. A free concentration of 20 drug molecules is considered, except for NA, which is set to 100, and for IFN+NA, with IFN = 10 and NA = 50 (half concentration for each drug). Black shows the absence of treatment (Control) for comparison. (**b**) The dynamics of complete virions released after adding different PS-targeting compounds. (**c**) Impact of CAMs on the probability of prevention of cccDNA formation over 10,000 simulations after viral entry, starting with the initial condition rcNC = 1. We only focus on the cccDNA formation step (Step 1; Figure 1) when one rcNC has entered the cell. α is the delivery rate of rcDNA to the nucleus, and we assume that the conversion rate of rcDNA to cccDNA is 1 h${}^{-1}$ (a=1 h${}^{-1}$). (**d**) Synergistic effect between CAM and compound 15 (C15), with treatment starting once the initial condition cccDNA = 1 is reached in the model. The average number of released complete virions when the concentration of each drug is equal to 10 molecules is shown for clarity. This figure is plotted using parameter values from Supplementary Table S1.

Simultaneous modelling of the steps of a viral life cycle targeted by different drugs permits assessment of their relative merits and synergies. For the two types of approved anti-HBV therapy, i.e., interferon (IFN)-based therapy (IFN) and nucleot(s)ide analogues (NAs), data on the clinical outcomes of therapy are available and can be used to validate the model. The levels of HBeAg protein, an indicator of active viral replication, and the HBV surface proteins (SVPs) are typically used as indicators of the severity of an infection. Based on these measures, IFN has been reported to perform better than NAs, and no synergistic effects between these two treatments were observed [2]. This is in excellent agreement with our model (Figure 5a). Even when the NA concentration (based on Tenofovir, the most efficient analogue) was set to five times that of IFN (using a free concentration of 20 molecules for this drug, which was added once the first cccDNA was formed), it still performed worse. The IFN+NA curve in Figure 5a shows the impact of using a free concentration of 50 molecules of NA plus 10 molecules of IFN (adding half the concentration of each drug to the system), with no significant synergistic effects between these drugs.

Figure 5 shows the impact of treatment options on the level of released complete virions. Supplementary Figure S11 indicates their impact on the level of released incomplete particles. This figure shows that IFN therapy, which targets the transcription process and release inhibitors, blocks the budding process, can reduce the level of incomplete particles in a single cell model slightly, but the other treatment options have no impact. However, by blocking the formation of new complete virions, these treatments will reduce the level of incomplete particles indirectly. The impact of this reduction could be studied in an intercellular model of HBV infection.

Given the success of the model in capturing the relative merits of known treatment options, we applied it to the additional antiviral therapies listed in [3] (Figure 2), which are currently at different stages of development. These include: Cp-assembly-directed CAMs (based on the most efficient variant (JNJ-827); HBsAg release inhibitors; and small interfering RNAs (siRNAs). Details of the modelling and published binding affinities used are given in the Supplementary Material, and results are shown in the context of virion concentration in the absence of treatment (Control). For siRNAs, different choices of cleavage rates and dissociation constants were explored (Supplementary Figure S7), assuming a binding affinity of 0.1 nM, corresponding to the smallest value known for any of the existing drugs to give this drug an advantage. We also chose a cleavage rate of 10 h${}^{-1}$; Supplementary Figure S7 shows that increasing the cleavage rate above 10 h${}^{-1}$ does not have a significant effect on the outcome for this drug. For release inhibitors, we assumed their efficacy to be equal to 95%, as a generous assumption. Even under these conditions, these drugs are outperformed by CAMs (Figure 5a). Geldanamycin (GA) is not included in the comparison, as it performed significantly worse than the other drugs. Despite an initial delay in virion release due to inhibition of P and pgRNA complex formation (Supplementary Figure S5a), it resulted in an increase in the number of virions released (Figure 3e). Similarly, targeting the HBx protein, which is presented as a possible future treatment option, does not appear to be a promising strategy (note that decreasing *b* is not effective; see Supplementary Figure S5). The assembly inhibitor CAM performs best, reducing the total number of complete virions to ∼3% of the Control.

Given the success of the NC assembly inhibitors, we explored an alternative strategy to block its formation. Recently, a series of small-molecular-weight compounds that bind to the three HBV PSs, inhibiting nucleocapsid assembly in vitro, have been identified. We modelled their action via the (un)binding to PSs using the experimentally measured affinities of these lead compounds for the three PSs (Supplementary Table S2). Figure 5b shows a comparison: Compound 15 (C15) performs best and is comparable with the outcome for the CAMs in Figure 5a. This compound binds with significant affinity to all three PSs, whilst the next best compound (C21) binds strongly to PS1 but very weakly to all others. This suggests that cooperativity between PSs in the pgRNA is important, making drugs capable of binding tightly to several PSs simultaneously highly effective.

Due to the antiviral effects of both CAMs and C15, we also explored synergies between these drugs. Rather than the full dose (20 molecules) for one of the drugs in isolation, as in Figure 5a,b, administering a lower dose of both (six molecules for each) achieves the same result (Figure 5d), suggesting a synergistic effect. In contrast, combinations of CAMs and PS-targeting drugs with other drug options, such as IFN, Tenofovir, release inhibitors, and siRNAs, did not show any significant synergistic effects. Supplementary Figure S12 shows that the impact of treatment starts at different times post-infection, indicating that nucleocapsid assembly inhibitors (CAM and C15) consistently perform better than other treatment options and exhibit synergistic effects between them.

cccDNA is an important marker of chronic liver disease caused by HBV. To compute the probability of cccDNA formation after viral entry in the drug-free case, we focused on the reactions of the cccDNA formation step (Step 1; Figure 1), which are rcDNA delivery to the nucleus (with rate α) and rcDNA conversion into cccDNA (with rate *a*). We did 10,000 simulations on this set of reactions for each value of α and *a* when one rcNC had entered the cell.Figure 4b shows the number of simulations out of 10,000 in which the cccDNA was not formed as a function of α and *a*. This figure indicates that, whilst the rcDNA conversion rate to cccDNA (*a*) has only minimal impact on cccDNA formation, the impact of the delivery rate of rcDNA to the nucleus (α) is significant for the outcome. CAMs can bind to, and thus disassemble, the nucleocapsid after cell entry before delivery of the rcDNA to the nucleus, thus reducing α. In particular, the disassembly rate ($k_{dis}$) has a significant impact on the prevention of cccDNA formation (Figure 5c). Figure 5c is plotted similarly to Figure 4b, but in the presence of CAMs. By contrast, using anti-host DNA factors (DDs) has minimal impact on the production of cccDNA unless they have almost unrealistically small $K_{d}$ ($K_{d}<1\mathrm{nM}$; Supplementary Figure S6), and they are therefore not likely to be as effective as CAMs in preventing cccDNA production. CAMs are therefore more promising routes to therapy than the DDs that are currently being discussed as possible therapeutic strategies.

## 4. Discussion

The in silico model of intracellular HBV infection dynamics introduced here is the most comprehensive model to date and contains reactions for all pathways targeted by either available anti-HBV drugs, or by proposed novel antiviral strategies that are alternative options. It therefore lends itself for a comparative analysis of different therapeutic strategies and can inform potential synergies.

Assessment of the impacts of different antiviral strategies [3,7] reveals that nucleocapsid assembly inhibitors, such as CAMs and PS-targeting drugs, are more promising than other treatment options. Interestingly, we observed a strong synergistic effect between CAMs and PS-targeting drugs, whilst other treatment options do not show comparable synergistic effects with nucleocapsid assembly inhibitors. This suggests that nucleocapsid assembly is a promising drug target, and could play an important role in achieving the World Health Organisation’s (WHO’s) goal of eliminating HBV by 2030 [3].

The presence of cccDNA is a marker of HBV infection and a target of curative strategies. We explored the consequences of therapeutic interventions preventing the formation of new cccDNA in the context of our model. Our results suggest that strategies targeting the delivery of the rcDNA into the nucleus are more effective than those targeting the conversion of rcDNA into cccDNA. Our model also reveals that Geldanamycin (GA), which targets the start of virion assembly (Step 5; Figure 1), is not a promising strategy because the total number of virions released can increase in the serum, despite initial delays in virion release. Similarly, our model demonstrates that targeting the HBx protein that promotes cccDNA activation and blocks its silencing (Step 2; Figure 1) does not perform as well as other drug strategies. It thus delivers a platform with which to assess the relative potential of different antiviral strategies and provides a pointer towards the most promising options for experimental development.

In contrast to continuous models, our discrete stochastic model permits tracking of particles at low concentrations during the kinetic phase, providing unprecedented insights into the production of different particle types at the early stages of an infection. In addition, this model revealed a previously unrecognised two-phase behaviour in the release of spherical SVPs. We established that it occurs for all biologically relevant conditions, suggesting that it is a biologically meaningful phenomenon. It is possible that the occurrence of high levels of spherical SVPs at the very start of an infection and again in a second phase after virion release has stalled could have consequences for the immune response. Our model provides the foundation for studying such aspects of infection dynamics by coupling the intracellular with an intercellular model in a multiscale approach. For parameters that were not measured in vivo, we used estimates based on in vitro studies and other arguments, as outlined in Table S1 and Section S3 in the Supplementary Material. Once such parameters become available, they can be used to improve the predictive power of the model. In any case, our intracellular model provides the first step in developing a multiscale within-host model to understand the immune response to HBV infections.

## Data Availability

Data is contained within the article and supplementary material.

## Supplementary Materials

The following are available online at https://www.mdpi.com/1999-4915/13/1/11/s1, Figure S1: The number of ribosomes (rib) plays an important role in the dynamics of the model, Figure S2: If the parameter has a biologically relevant value, Figure S3: The binding rate of RNA Pol II to cccDNA does not have any impacts on the dynamics of the model, Figure S4: The probability of prevention of cccDNA formation with anti-host DNA repairfactors as a function of the parameter a and the binding anity (Kd) of this drug, Figure S5: The number of complete virions released with RNA interference treatment and parameter values from Table S1 as a function of the binding anity (Kd) and the cleaving rate (kcleave) of this drug, Figure S6: The number of complete virions released with CAM treatment and parameter values from Table S1, Figure S7: The number of complete virions released with parameter values from Table S1, Figure S8: The average of cumulative populations of proteins inside the cell over 100 simulations, Figure S9: The dynamics of incomplete particles released with different treatment options after cccDNA formation, Figure S10: The number of complete virions released with starting treatment at different times post-infection and parameter values from Table S1, Figure S11: The HBV life cycle. Step 0: Viral entry is mediated by the sodium-taurocholate co-transporting polypeptide (NTCP) receptor, Figure S12: Current and potential future drugs for the treatment of HBV. Table S1: Table of parameter values, Table S2: Table of dissociation constants of PS-targeting compounds, Equations S1: An In Silico Model of Intracellular HBV Infection Dynamics, Equations S2: Eects of Drug Treatments, Equations S3: Parameter Estimation.
