# Supplementary material for: An Intracellular Model of Hepatitis B Viral Infection: An In Silico Platform for Comparing Therapeutic Strategies
Source: Viruses. 2020 Dec 23;13(1):11. doi: 10.3390/v13010011 (PMC7823939; doi:10.3390/v13010011)
Supplement: Supplementary file 1 [file viruses-13-00011-s001.pdf]

An Intracellular Model of Hepatitis B Viral Infection: An *In Silico* Platform to Compare Therapeutic Strategies

Farzad Fatehi, Richard J Bingham, Eric C Dykeman, Nikesh Patel, Peter G Stockley* and Reidun Twarock*

*Correspondence: rt507@york.ac.uk, p.g.stockley@leeds.ac.uk

Supplementary Material

S1 An *In Silico* Model of Intracellular HBV Infection Dynamics

Here, we provide a full description of all the steps of the model, as shown in Figure S13, with their equations:

Step 1—Viral entry and cccDNA production: After entering into a susceptible host cell (via a hepatic bile acid transporter receptor, the sodium taurocholate cotransporting polypeptide (NTCP) [1]), the virion (rcNC) releases its cargo with rate α into the nucleus [2]. Inside the virion, the genome is stored as relaxed circular DNA (rcDNA). In the nucleus, rcDNA converts into a covalently closed circular DNA (cccDNA) via cellular enzymes that are normally involved in base excision repair (BER) of DNA damage [3, 4], essentially recognising the rcDNA as "damaged" DNA inside the cell nucleus. Since the enzymes acting as catalysts in this reaction are readily available in the cell and are therefore not rate limiting, we model this as a conversion with rate a. This reaction is a coarse-grained model for the following biological step: An endonuclease, such as XPG, cleaves the viral DNA strands close to their $5'$ ends to remove both the polymerase (P) protein and the capped RNA. Additionally, one or both of the short terminal redundancies (r), which are around nine nucleotides and are located at the $3'$ and $5'$ ends of the minus strand, are removed. Then, a cellular DNA polymerase, such as DNA polymerase κ, and either ligase I or ligase III attach and join the $5'$ and $3'$ ends of the two DNA strands. Natural degradation of cccDNA and rcDNA is modelled at rate μ [5]. The reactions used to model step 1 are therefore:

$$\text{rcNC} \xrightarrow{\alpha} \text{rcDNA}, \quad \text{rcDNA} \xrightarrow{a} \text{cccDNA},$$
$$\text{rcNC} \xrightarrow{\mu_R} 0, \quad \text{cccDNA} \xrightarrow{\mu} 0, \quad \text{rcDNA} \xrightarrow{\mu} 0.$$

Step 2—Epigenetics of cccDNA: An important factor in liver disease caused by HBV infections is the cccDNA. This molecule is organised into mini-chromosomes with host cell histones and potentially other host and viral proteins [6, 7]. Therefore, transcription from cccDNA is subject to epigenetic regulation, with numerous options for dynamic epigenetic control of cccDNA transcriptional activity [8, 9, 10]. In the absence of the X protein (HBx), which gets produced by the cccDNA over the course of the infection, cccDNA appears to be rapidly silenced [11]. The X protein promotes the de-silencing of cccDNA, and blocks the silencing of cccDNA [12]. Therefore, this process can be modelled akin to gene expression models, where a gene is activated and deactivated [13]. For this, we set λ_{off} and λ_{on} to be the silencing and de-silencing rates, respectively. As increasing the amount of the X protein reduces the silencing rate and increases the de-silencing rate of cccDNA, we assume that cccDNA becomes silenced and de-silenced at rates $\lambda_{off}/(1 + s\overline{\text{X}})$ and $\lambda_{on}(1 + s\overline{\text{X}})$, respectively, using the constant s to indicate the efficiency of this process:

$$\text{cccDNA} \underset{\lambda_{on}(1+s\overline{\mathrm{X}})}{\overset{\frac{\lambda_{off}}{1+s\overline{\mathrm{X}}}}{\rightleftharpoons}} \text{silenced cccDNA.}$$

$\overline{\mathrm{X}}$ indicates the amount of HBx protein in this model.

Step 3—Transcription of cccDNA: At this stage, the cccDNA is used by the host RNA polymerase II (RNA Pol) as a template to transcribe all viral RNAs. This includes the pgRNA (also called the C mRNA [14]) for both the core (C) and polymerase (P) proteins; X mRNA for the X protein; LS mRNA for the L surface protein; S mRNA for the M and S surface proteins; and PreC mRNA for the precore protein. The production of specific mRNAs happens via binding of RNA Pol II to cccDNA. The production of HBV RNAs is modelled as follows, where RNA Pol II and the cccDNA bind together and produce all mRNAs. We assume that there are three different binding rates (b_1, b_2, b_3). Thus, we have:

$$\begin{aligned}
&\text{cccDNA} + \text{RNA Pol} \xrightarrow{b_1} \text{cccDNA:Pol1},\\
&\text{cccDNA} + \text{RNA Pol} \xrightarrow{n_{Ld}b_1} \text{cccDNA:Pol2},\\
&\text{cccDNA} + \text{RNA Pol} \xrightarrow{n_{Ld}b_1} \text{cccDNA:Pol3},\\
&\text{cccDNA} + \text{RNA Pol} \xrightarrow{b_2} \text{cccDNA:Pol4},\\
&\text{cccDNA} + \text{RNA Pol} \xrightarrow{b_3} \text{cccDNA:Pol5},\\
&\text{cccDNA:Pol1} \xrightarrow{c_1} \text{pgRNA} + \text{RNA Pol} + \text{cccDNA},\\
&\text{cccDNA:Pol2} \xrightarrow{c_2} \text{LS mRNA} + \text{RNA Pol} + \text{cccDNA},\\
&\text{cccDNA:Pol3} \xrightarrow{c_3} \text{S mRNA} + \text{RNA Pol} + \text{cccDNA},\\
&\text{cccDNA:Pol4} \xrightarrow{c_4} \text{X mRNA} + \text{RNA Pol} + \text{cccDNA},\\
&\text{cccDNA:Pol5} \xrightarrow{c_5} \text{PreC mRNA} + \text{RNA Pol} + \text{cccDNA}.
\end{aligned} \tag{S1}$$

Since the level of surface protein is higher than the HBV DNA level, we assume the binding rates that lead to the production of the LS and S mRNAs to be n_{Ld} times larger [5], and $b_1 = b_2 = b_3 = b$. The parameters $c_1, c_2, ...c_5$ are elongation rates.

Step 4—Viral protein synthesis: All viral mRNAs have to be exported from the nucleus to the cytoplasm in order to be translated [2]. Once a free ribosome successfully binds to mRNA at rate d, a protein is produced and the ribosome releases the mRNA [15]. The pgRNA is bicistronic and produces the C and P proteins. Thus, when the ribosome binds to pgRNA, it can produce either the C or P protein. This has been modelled as follows:

$$\begin{aligned}
&\text{Rib} + \text{pgRNA} \xrightarrow{d} \text{pg:Rib}_1,\\
&\text{pg:Rib}_1 \xrightarrow{e_1} \text{P} + \text{Rib} + \text{pgRNA},\\
&\text{Rib} + \text{pgRNA} \xrightarrow{n_c d} \text{pg:Rib}_2,\\
&\text{pg:Rib}_2 \xrightarrow{e_2} \text{C} + \text{Rib} + \text{pgRNA},
\end{aligned} \tag{S2}$$

where e_1 and e_2 are translation rates. As production of a virion needs more C proteins than polymerase, we assume that the pg:Rib$_2$ complex is produced at an n_c-fold higher rate than pg:Rib$_1$.

A similar argument applies to the other mRNAs. Hence, we have the following equations:

$$\begin{aligned}
&\text{Rib} + \text{LS mRNA} \xrightarrow{n_{Lm}d} \text{LS:Rib},\\
&\text{LS:Rib} \xrightarrow{e_3} \text{L} + \text{Rib} + \text{LS mRNA},\\
&\text{Rib} + \text{S mRNA} \xrightarrow{n_{Lm}d} \text{S:Rib}_1,\\
&\text{S:Rib}_1 \xrightarrow{e_4} \text{M} + \text{Rib} + \text{S mRNA},\\
&\text{Rib} + \text{S mRNA} \xrightarrow{n_s n_{Lm}d} \text{S:Rib}_2,\\
&\text{S:Rib}_2 \xrightarrow{e_5} \text{S} + \text{Rib} + \text{S mRNA},\\
&\text{Rib} + \text{X mRNA} \xrightarrow{d} \text{X:Rib},\\
&\text{X:Rib} \xrightarrow{e_6} \text{X} + \text{Rib} + \text{X mRNA},\\
&\text{Rib} + \text{PreC mRNA} \xrightarrow{d} \text{PreC:Rib},\\
&\text{PreC:Rib} \xrightarrow{e_7} \text{PreC} + \text{Rib} + \text{PreC mRNA},
\end{aligned}$$

where $e_3, e_4, ..., e_7$ are translation rates. We assume the binding rates that lead to the production of surface proteins to be n_{Lm} times larger [5]. Similarly to pgRNA, S mRNA is also bicistronic, and we assume the S:Rib$_2$ complex to be produced at an n_s-fold higher rate because S protein is the most common protein in the viral envelopes and is known to result in production of multiple non-viral particles [16].

Note, in the protein production reactions, that these proteins are produced as monomers. Subsequently, they bind to form dimers, which act as building blocks. However, for simplicity, we assume in this model that these proteins are already in dimeric form, as removing this step does not affect model behaviour.

The following equations describe the degradation of mRNAs and proteins:

$$\begin{aligned}
&\text{pgRNA} \xrightarrow{d_m} 0, \quad \text{LS mRNA} \xrightarrow{d_l} 0, \quad \text{S mRNA} \xrightarrow{d_s} 0, \quad \text{X mRNA} \xrightarrow{d_x} 0,\\
&\text{PreC mRNA} \xrightarrow{d_p} 0, \quad \text{P} \xrightarrow{\delta_p} 0, \quad \text{C} \xrightarrow{\delta_c} 0, \quad \text{L} \xrightarrow{\delta_l} 0, \quad \text{M} \xrightarrow{\delta_m} 0, \quad \text{S} \xrightarrow{\delta_s} 0,\\
&\text{X} \xrightarrow{\delta_x} 0, \quad \text{PreC} \xrightarrow{\delta_{pc}} 0.
\end{aligned}$$

These degradation rates have been computed based on the half-lives of these mRNAs and proteins.

Step 5—Capsid assembly initiation: Assembly of HBV depends on the formation of a ribonucleoprotein (RNP) complex formed from P and pgRNA [2, 17]. A short structured RNA signal located at the 5′ end of the pgRNA, called ε, was identified as the RNA packaging signal mediating the packaging of pgRNA into the nucleocapsid (NC) [18, 19]. ε was later found to be recognised specifically by the P protein, not C, and P and pgRNA packaging are mutually dependent [20, 21]. The formation of this RNP complex depends on cellular factors, which include the heat shock protein 90 (Hsp90), and also require ATP hydrolysis and the function of p23, an identified chaperone partner for Hsp90 [3, 17]. p23 can bind to the polymerase independently of Hsp90. The binding of Hsp90 and p23 to the polymerase is not sufficient to enable the polymerase to bind ε , but the Hsp90–p23 interaction is critical for the formation of the viral RNP complex (binding of P to the pgRNA) [17]. Since Hsp90 and p23 are cellular proteins, whose production is independent of the virus, we assume that they are available in sufficient quantities and are therefore not rate limiting. Thus, in the mathematical model, we only represent the binding of pgRNA and P with rate g, with the impact of these different proteins contained implicitly in the rate:

$$\text{pgRNA} + \text{P} \xrightarrow{g} \text{RNP}.$$

Step 6a—Capsid assembly: The RNP complex is ready to be encapsidated. There are three PSs (denoted as PS1, PS2, and PS3) in the pgRNA with affinity for the capsomer (a CP dimer) [22]. Comparing the assembly of HBV in the absence and presence of multiple copies of these PSs suggests that they are important for efficient capsid assembly [22]. We therefore assume that all three PSs interact with CP. In particular, denoting the RNP complex by R, and indicating by C$_j$, $j = 1, 2, 3$ that PSj is bound to C, we have 12 reactions between complexes in which different combinations of these PSs are bound to C (Figure S1). Black arrows represent the binding/unbinding of C to/from the R.

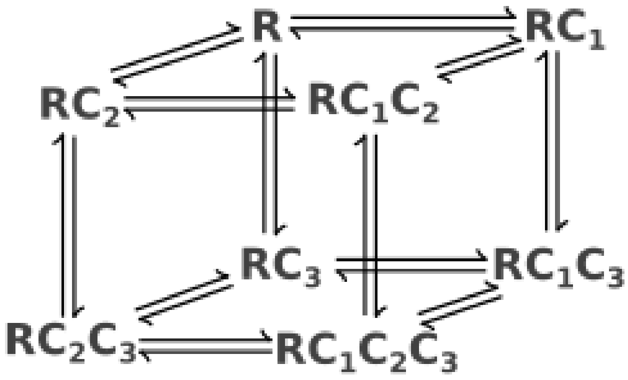

Figure S1: All the possible reactions of the RNP complex (R) with C proteins.

Once the complex $RC_1C_2C_3$ is formed, more Cs are recruited to build the full capsid according to the following reactions:

$$
\begin{aligned}
&\mathrm{RC_1C_2C_3} + \mathrm{C} \xrightarrow{\kappa} \mathrm{R{:}4C},\\
&\mathrm{R{:}jC} + \mathrm{C} \xrightarrow{\kappa} \mathrm{R{:}(j{+}1)C}, \quad \mathrm{j} = 4, \dots, 118,\\
&\mathrm{R{:}119C} + \mathrm{C} \xrightarrow{\kappa} \mathrm{pgNC},
\end{aligned}
$$

where κ is an average binding rate of C to the growing capsid shell, and pgNC denotes a fully formed capsid containing pgRNA, called the immature nucleocapsid.

Step 6b—Empty capsid assembly: It has been discovered recently that infected cells also produce empty virions, i.e., empty viral capsids enclosed by surface proteins [14]. Creation of an empty virion is made of two steps: first, the nucleation step where C proteins bind together at a slower rate [23, 24]. We model this step as follows:

$$
\mathrm{C} + \mathrm{jC} \xrightarrow{\kappa_n} \mathrm{(j{+}1)C}, \quad \mathrm{j} = 1, \dots, (nuc - 1),
$$

where *nuc* is the number of core proteins that are needed for creating the nucleation complex and k_n is the forward rate for the nucleation reactions.

The second step is the elongation step where the remaining C proteins assemble at the faster rate κ_2 to form an empty nucleocapsid (emNC):

$$
\begin{aligned}
&\mathrm{C} + \mathrm{jC} \xrightarrow{\kappa} \mathrm{(j{+}1)C}, \quad \mathrm{j} = nuc, \dots, 118,\\
&\mathrm{119C} + \mathrm{C} \xrightarrow{\kappa} \mathrm{emNC}.
\end{aligned}
$$

In this model, we assume that empty capsids are made of 120 C proteins following a $T = 4$ architecture, ignoring the $T = 3$ particles that occur in only 5% of cases [22].

Step 7—Reverse transcription: The reverse transcription of the pgRNA starts at this stage. In $\sim 90\%$ of cases, this process leads to the synthesis of rcDNA, and in the remaining 10%, to double-stranded linear DNA (dslDNA) [25]. Therefore, we have

$$
\begin{aligned}
&\mathrm{pgNC} \xrightarrow{0.9s_1} \mathrm{rcNC},\\
&\mathrm{pgNC} \xrightarrow{0.1s_1} \mathrm{dslNC},\\
&\mathrm{pgNC} \xrightarrow{\mu_R} 0,\\
&\mathrm{rcNC} \xrightarrow{\mu_R} 0,\\
&\mathrm{dslNC} \xrightarrow{\mu_R} 0,
\end{aligned}
$$

where rcNC and dslNC indicate nucleocapsids containing rcDNA and dslDNA, respectively. These are called the mature nucleocapsids.

Step 8—Intracellular cccDNA amplification: rcNC and dslNC can deliver their contents into the nucleus to amplify the cccDNA reservoir [2]. The viral surface protein L regulates cccDNA amplification, such that during the early stage of infection, when L protein levels are low, the rcDNA is recycled [2, 14]. Murray and Goyal [5] introduced this effect into their

model through the term $e^{-\lambda \mathrm{L}}$, where λ^{-1} specifies the average level of the L protein [26]. Thus, we have the following equations:

$$\begin{aligned} \text{rcNC} &\xrightarrow{te^{-\lambda \mathrm{L}}} \text{rcDNA}, \\ \text{dslNC} &\xrightarrow{te^{-\lambda \mathrm{L}}} \text{dslDNA}, \end{aligned} \tag{S3}$$

where t is the export rate.

Note: There is another step in the viral life cycle called HBV DNA integration, where the dslDNA can be integrated into the host cell genome, where it acts as a template for LS mRNA, S mRNA, and X mRNA. This integration occurs once in any $\sim 10^5-10^6$ infected cells [25]. Alternatively, dslDNA can be circularised via the non-homologous end-joining (NHEJ) DNA repair pathway, supported by the involvement of Ku80, into cccDNA molecules [27]. However, as NHEJ is error-prone, many of these molecules are functionally defective [12]. As Ku80 does not get consumed during this reaction [28], we do not model it explicitly. Therefore, we have the following reactions:

$$\begin{aligned} &\text{dslDNA} \xrightarrow{y_1} \text{dslDNAint}, \\ &\text{dslDNAint} + \text{RNA Pol} \xrightarrow{b_1} \text{int:Pol}, \\ &\text{int:Pol} \xrightarrow{c_2} \text{LS mRNA} + \text{RNA Pol} + \text{dslDNAint}, \\ &\text{int:Pol} \xrightarrow{c_3} \text{S mRNA} + \text{RNA Pol} + \text{dslDNAint}, \\ &\text{int:Pol} \xrightarrow{c_4} \text{X mRNA} + \text{RNA Pol} + \text{dslDNAint}, \\ &\text{dslDNA} \xrightarrow{y_2} \text{cccDNA}, \\ &\text{dslDNA} \xrightarrow{\mu_d} 0. \end{aligned}$$

As the production rate of dslNC is much lower than that of rcNC (less than 10%) and the integration occurs once in any $\sim 10^5 - 10^6$ infected cells, and since the NHEJ is error prone, many of cccDNAs produced from dslDNA are functionally defective, and we do not integrate this step into the model and only consider the rcNCs.

Step 9a—Secretion of complete virions: When the level of the L protein is sufficiently high, rcNCs acquire the viral surface proteins and are secreted outside the cell via the cellular secretory pathway [2]. The release of infectious virions occurs via multivesicular bodies (MVBs) using the ESCRT (endosomal sorting complex required for transport) machinery [29, 30]. HBV virions bud into late endosomes or MVBs by utilising ESCRT/Vps4B (vacuolar protein sorting 4B) functions and exit the cell by the exosome pathway [31].

The estimated number of surface protein subunits (monomers) in viral particles is 210–270 subunits. Therefore, it has been suggested that there is a 1:1 stoichiometry between the core and the surface subunits [32, 33]. As surface proteins also occur as dimers [31], this means that we need 120 surface protein dimers to complete a virion. As the ratio of L, M, and S proteins has not been measured in infectious virions yet or in filaments, which we will discuss later, the ratio of L, M, and S is 1:1:4 [34], and we assume that an infectious virion contains L, M, and S proteins with the same ratio (1:1:4). Since rcNCs recycle their contents at rate $e^{-\lambda \mathrm{L}}$, which is between zero and one, they are released at rate $1 - e^{-\lambda \mathrm{L}}$ [5]. As a complete virion collects the surface proteins during budding, we consider it as one reaction in the model. Hence, we have:

$$\text{rcNC} + 80\text{S} + 20\text{M} + 20\text{L} \xrightarrow{t(1-e^{-\lambda \mathrm{L}})} \text{complete virion}.$$

Step 9b—Secretion of RNA-containing particles: It has been discovered recently that infected cells also secrete, albeit at a low level, RNA-containing particles [14, 35]. The level of these particles in a patient is around 0.1%–1% of the HBV DNA level in the absence of antiviral treatment [36, 37, 38, 39, 40]. Akin to secretion of complete virions, it can be modelled as follows:

$$\text{pgNC} + 80\text{S} + 20\text{M} + 20\text{L} \xrightarrow{t_1(1-e^{-\lambda \mathrm{L}})} \text{RNA-containing particle}. \tag{S4}$$

Step 9c—Secretion of empty virions: Like complete virions, we assume that the ratio of L, M, and S proteins in these particles is 1:1:4. These are typically found at 100-fold higher

levels than infectious virions [14]. Akin to secretion of complete virions, this can be modelled as follows:

$$\text{emNC} + 80\text{S} + 20\text{M} + 20\text{L} \xrightarrow{t(1-e^{-\lambda \text{L}})} \text{empty virion}. \tag{S5}$$

In addition to complete, RNA-containing, and empty virions, infected cells produce non-infectious viral particles that contain no nucleocapsid (viral genome) and contain only the outer envelope layer of the virion (surface protein) [2]. These so-called subviral particles (SVPs) include the classical octahedra spheres containing 48 S proteins, with a diameter of about 20 nm, as well as filaments with the same diameter, but with variable length [32, 16]. SVPs are typically found at 1000–100,000-fold higher levels than infectious virions [41, 14].

Step 9d: Secretion of filaments: Tubular filaments are a form of SVP that are also called L-rich subvirals [31]. These particles are estimated to contain L, M, and S proteins in a ratio of 1:1:4 [34]. Experimental groups have suggested that these empty filaments are formed via conventional tubular budding at the membrane of the same compartments for the release of infectious virions [42, 30]. Since the secretion of filaments and infectious virions happens through the same compartment, our assumption about the ratio of L, M, and S proteins for infectious virions (1:1:4) is reasonable. We assume that filaments, like infectious virions, contain 120 surface proteins on average. Therefore, we have:

$$80\text{S} + 20\text{M} + 20\text{L} \xrightarrow{t_3(1-e^{-\lambda \text{L}})} \text{filament}, \tag{S6}$$

where t_3 is the secretion rate of filamentous SVP.

Step9e—Secretion of spheres: Spherical particles are the other form of SVP. These particles are octahedral, i.e., they are made of 48 S protein dimers. Spherical SVPs have a different secretion pathway from that of infectious virions [31]. To create these particles, S proteins that are attached to the membrane of the endoplasmic reticulum (ER) initially self-assemble in a filamentous form with a diameter of 20 nm in the perinuclear space, which forms part of the ER. Then, these filaments of about 0.2–0.3 µm in diameter are transported via ER-derived vesicles to the rough ER–Golgi intermediate compartment (ERGIC) where they are unpacked, relaxed, and converted into spheres, and then exit the cell [43, 31, 16].

Since S proteins contributing to these filaments are inaccessible to NCs and spherical SVPs are released in much higher numbers than infectious virions, in order to simplify our model, we assume that as soon as 48 S proteins are assembled, they are released as a spherical particle, rather than creating larger filaments that are then divided into several particles. Although this assumption is a simplification, it does not affect the overall outcome of the model, as filaments keep budding spheres at a fast rate, which can be modelled by choosing the right value for the budding rate.

It has been reported that a small proportion of L protein results in the production and secretion of spherical SVPs, while a larger amount of L protein results in the formation and secretion of filaments and infectious virions [44, 26, 42]. Hence, we consider the following reactions:

$$\begin{aligned} &\text{S} + \text{S} \xrightarrow{\kappa e^{-\lambda \text{L}}} 2\text{S}, \\ &\text{jS} + \text{S} \xrightarrow{\kappa} (\text{j+1})\text{S}, \quad \text{j} = 2, \dots, 47, \\ &48\text{S} \xrightarrow{t_2} \text{sphere}, \end{aligned} \tag{S7}$$

where the impact of L protein is added to the initiation reaction, κ is the binding rate of S proteins, and t_2 is the secretion rate of spherical SVPs.

S2 Effects of Drug Treatments

In this section, we focus on the current and future treatment options for chronic hepatitis B. Figure S14 shows all the treatment options and the process that they attack. Below, we discuss details for each drug and introduce our mathematical models for their impacts.

Interferon-α: Interferon (IFN)-based therapy includes stand-alone IFN-α or pegylated interferon-α (Peg-IFN-α) [45]. IFN-α produces its antiviral effect via the activation of natural killer (NK)/NKT cells, which can be studied in an intercellular model [45], via direct

suppression of RNA and protein production in infected cells and degradation of cccDNA via the activation of APOBEC3A in infected cells [46, 47, 48]. IFN-α results in cccDNA-bound histone hypoacetylation as well as active recruitment to the cccDNA of transcriptional corepressors. IFN-α treatment also reduces binding of transcription factors to active cccDNA [9]. Thus, in the model, we consider that IFN-α binding silences the cccDNA and IFN-αincreases the degradation rate of cccDNA. Hence, the additional reactions have the form:

$$\mathrm{IFN} + \mathrm{cccDNA} \underset{I_{unbind}}{\overset{I_{bind}}{\rightleftharpoons}} \mathrm{ccc{:}IFN},$$
$$\mathrm{cccDNA} \xrightarrow{\mu(1+\psi \mathrm{IFN})} 0,$$

where IFN is the amount of IFN-α, ψ is the efficacy of this drug in degrading of the cccDNA, and I_{bind} and I_{unbind} are the binding and unbinding rates of IFN-α, respectively. However, this treatment, which can lead to complete viral suppression in approximately 25% of patients, is associated with significant side effects [49, 50].

Geldanamycin: Geldanamycin (GA) is known to bind Hsp90 and to disrupt Hsp90-p23 complexes [51, 52, 53]. The binding of GA to P happens through Hsp90 and inhibits the formation of RNP [17]. As we are assuming that Hsp90 and p23 are not rate limiting, we can ignore them in the model. We model the GA binding to P to create the P:GA complex, which cannot bind to the pgRNA. Therefore, we have the following reactions:

$$\mathrm{P} + \mathrm{GA} \underset{k_{unbind}}{\overset{k_{bind}}{\rightleftharpoons}} \mathrm{P{:}GA},$$

where k_{bind} and k_{unbind} indicate the binding and unbinding rates of GA, respectively.

Nucleos(t)ide analogues: Nucleos(t)ide analogues (NAs), such as lamivudine, adefovir, entecavir, tenofovir, telbivudine, famciclovir, and clevudine [45], incorporate into growing DNA strands and inhibit the synthesis of mature from immature nucleocapsids [12, 35]. In order to model the effects of these drugs, we assume that NAs can bind to the pgNC to stop the process of reverse transcription; hence:

$$\mathrm{pgNC} + \mathrm{NA} \underset{l_{unbind}}{\overset{l_{bind}}{\rightleftharpoons}} \mathrm{pgNC{:}NA},$$
$$\mathrm{pgNC{:}NA} + 80\mathrm{S} + 20\mathrm{M} + 20\mathrm{L} \xrightarrow{t_1(1-e^{-\lambda \mathrm{L}})} \mathrm{RNAVirion},$$

where l_{bind} and l_{unbind} are the binding and unbinding rates of this drug to pgNC, respectively.

Although NA administration induces a strong viral suppression in the majority of patients, NAs usually have to be taken lifelong to prevent relapse [50, 49]. Despite viral suppression, a functional cure is achieved in only 10% of treated patients after a five-year follow up [50, 49].

PS-targeting drug: The packaging-signal-targeting drug occupies PSs and does not let C proteins bind to the PSs. Thus, these drugs disrupt the formation of the nucleocapsid and are considered as capsid inhibitors [35]. Peter G. Stockley's lab at the University of Leeds, in collaboration with the National Institutes of Health (NIH) USA and Imperial College London, has discovered some compounds that can bind to the PSs to disrupt virus assembly. A list of these compounds, whose impact on the inhibition of HBV has been tested *in vivo*, together with their binding affinities for all three PSs, is presented in Table S2 (Nikesh Patel, University of Leeds, private communication). The following diagram shows all possible reactions.

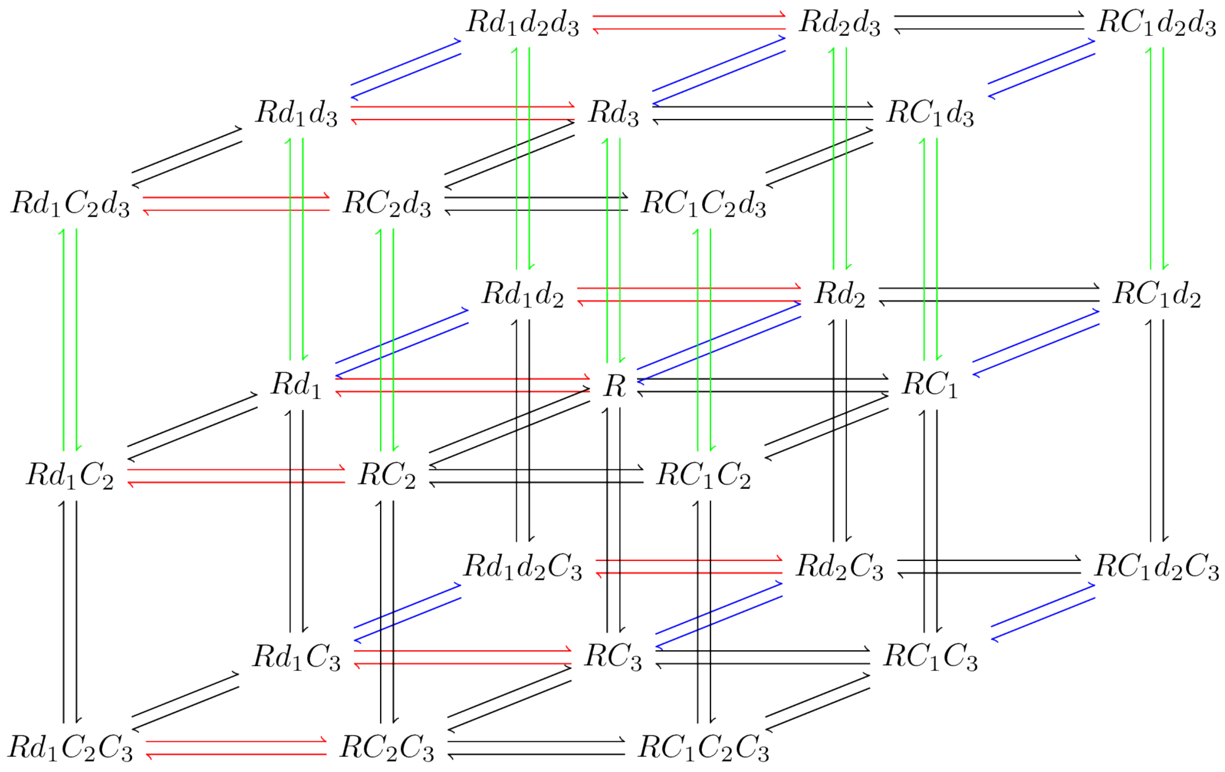

Figure S2: All the possible reactions of the RNP complex (R) with C proteins and PS-targeting drugs.

Here, the indices correspond to the packaging signal sites that are occupied by the drug (d) or capsid protein (C). For example, $RC_1C_2d_3$ indicates C binding to PSs at sites 1 and 2, and a drug bound to PS site 3. Red, blue, and green arrows indicate the binding/unbinding of the drug to/from the PS1, PS2, and PS3, respectively.

Core protein targeting drugs: Molecules targeting the core protein are named capsid assembly modulators (CAMs) or core protein allosteric modulators (CpAMs). These molecules bind to C protein and alter the kinetics and pathway of C protein assembly [54, 55]. This either leads to the formation of morphologically intact empty capsids (class I mechanism of action (MoA) compounds) or aberrant capsids devoid of pgRNA (class II MoA compounds) [56]. These drugs can inhibit formation of cccDNA due to an accelerated breakdown of capsids in the cytoplasm [57, 58]. It has been shown that elongation of positive-stranded DNA induces structural changes in the nucleocapsid, which cause mature nucleocapsid to bind CAMs and trigger capsid disassembly [54]. Moreover, CAMs efficiently inhibit replication of HBV mutants resistant to NAs [36, 59] and are active against multiple HBV genotypes [56].

To date, several CAMs are in preclinical evaluations or have entered clinical trials. Lahlali et al. [58] studied the impact of novel CAMs JNJ-827 and JNJ-890. They observed that JNJ-827 and JNJ-890 are class I and II, respectively. JNJ-827 and JNJ-890 are potent inhibitors of HBV replication with respective half-maximal effective concentrations of 4.7 and 66 nM, respectively.

Since this drug (CAM) can bind to core proteins and alter their assembly pathway, we add the following reactions regarding binding and unbinding of CAMs to core proteins:

$$\mathrm{C} + \mathrm{CAM} \underset{C_{unbind}}{\overset{C_{bind}}{\rightleftharpoons}} \mathrm{C{:}CAM},$$

where C_{bind} and C_{unbind} are the binding and unbinding rates of this drug to C protein, respectively.

The C:CAM complexes can bind together to form capsids. If the drug is class I, they form morphologically intact capsids. Therefore, 120 of them bind together, at a faster rate than C–C interactions (κ), to form empty capsids, and we assume that these capsids are secreted like the empty virions modelled above. Hence, we have:

$$\text{C:CAM} + \text{jC:CAM} \xrightarrow{\kappa_{nc}} \text{(j+1)C:CAM}, \quad \text{j} = 1, \ldots, (nuc - 1),$$
$$\text{C:CAM} + \text{jC:CAM} \xrightarrow{\kappa_1} \text{(j+1)C:CAM}, \quad \text{j} = nuc, \ldots, 118,$$
$$\text{119C:CAM} + \text{C:CAM} \xrightarrow{\kappa_1} \text{emNC:CAM},$$
$$\text{emNC:CAM} + \text{80S} + \text{20M} + \text{20L} \xrightarrow{t(1-e^{-\lambda \text{L}})} \text{emptyVirion},$$

where nuc is the number of core proteins that are needed for composing the nucleating structure [23, 60], k_{nc} is the forward rate for the nucleation reactions, and κ_1 is the forward rate for the elongation reactions.

As suggested in [54], CAMs can bind to rcNCs and trigger their disassembly followed by degradation of the rcDNA. We also assume that capsid proteins are partially assembled and therefore not available for buildup of new capsids; hence, we only include a reaction that results in CAM becoming available after dissociation, as follows:

$$\text{rcNC} + \text{CAM} \underset{C_{unbind}}{\overset{C_{bind}}{\rightleftharpoons}} \text{rcNC:CAM},$$
$$\text{rcNC:CAM} \xrightarrow{k_{dis}} \text{CAM},$$

where k_{dis} indicates the disassembly rate.

If the drug is class II, its binding to C causes the formation of aberrant capsids. In this case, for simplicity, we assume that aberrant capsids contain 120 C:CAM on average, and we model them in the same way, but we assume that they are degraded rather than released, as the effect on the overall infection dynamics is equivalent in both cases.

Note that we focus the model on a more efficient version of CAMs, i.e., JNJ-827, which is class I and has a smaller half-maximal effective concentration.

Novel therapeutic strategies: Biologists have proposed some new potentially curative therapies for chronic hepatitis B that are either evaluated in preclinical models or are in the first phases of clinical development [12, 35]. Below, we discuss details for each drug and introduce our mathematical models for their impacts.

Entry inhibitors: These drugs reduce the infection rate of target cells [35]. The impact of these drugs has to be studied in an intercellular model, which we will consider as a later stage.

Anti-host DNA repair factors: These drugs inhibit the conversion of rcDNA into cccDNA [12] by attacking the DNA repair factors. In our model, we assume that these factors are not rate limiting. Therefore, we ignore them in the model, and consider drugs (DDs) binding to rcDNA to create the rc:DD complex, thus preventing conversion into cccDNA:

$$\text{rcDNA} + \text{DD} \underset{m_{unbind}}{\overset{m_{bind}}{\rightleftharpoons}} \text{rc:DD},$$

where m_{bind} and m_{unbind} are the binding and unbinding rates of this drug, respectively.

Targeting cccDNA and HBx protein: It has been suggested that research directed towards elimination of cccDNA or permanently silencing cccDNA transcription should be prioritised [35]. For example, as X protein is necessary to counteract the silencing of cccDNA, it could be a target for direct-acting antivirals [12]. Therefore, these drugs increase and decrease the silencing and de-silencing rates of cccDNA, respectively. Studying how the intracellular dynamics of HBV change with the parameters s, λ_{off}, and λ_{on} reveals the impact of this treatment. Our numerical simulations show that increasing λ_{off} and decreasing λ_{on} are not effective treatment options. Therefore, targeting the HBx protein is not effective.

Parameter b (binding rate of RNA Pol II and cccDNA) does not have any impact on the number of complete and empty virions (Supplementary Figure S5). However, decreasing this parameter decreases the number of SVPs. This figure suggests that, for $b > 0.5$, the model always shows the same dynamic. In addition, Supplementary Fig. S3 shows that the number of RNA Pol II does not have any impacts on the dynamics of the model. However, these parameters can play an important role on the dynamics of the model when we add a treatment that attacks the cccDNA. This shows that measuring the amount of RNA Pol II in the nucleus and its binding rate to the cccDNA can help us in developing an effective treatment that attacks the cccDNA. These drugs have been argued as a possible future

treatment [35], and our model indicates that the efficacy of these drugs depends on the amount of RNA Pol II and its binding rate.

Using RNA interference: Post-transcriptional control of cccDNA or integrated HBV DNA expression can be achieved using RNA interference [35], which is incorporated into an RNA-induced silencing complex (RISC) that directs degradation of mRNAs. Therefore, this treatment increases the degradation rate of mRNAs, and its effect can be modelled as follows:

$$\text{pgRNA} + \text{siRNA} \underset{R_{unbind}}{\overset{R_{bind}}{\rightleftarrows}} \text{pg:siRNA},$$
$$\text{pg:siRNA} \xrightarrow{k_{cleave}} \text{siRNA},$$

where siRNA is the number of small interfering RNAs, R_{bind} is the binding rate of the interfering RNA to the pgRNA, and k_{cleave} is the cleaving rate, which leads to the degradation of pgRNA.

Inhibitors of viral and SVP release: These drugs target the viral and SVP egress, i.e., these drugs reduce the rates t, t_1, t_2, and t_3. Let us assume that η be the efficacy of these drugs, which is a number between 0 and 1. Then, one can represent the effect of this treatment by modifying the release rates to $(1-\eta)t$, $(1-\eta)t_1$, $(1-\eta)t_2$, and $(1-\eta)t_3$. These drugs can show a promising effect if η is high enough ($\eta \geq 0.98$).

S3 Parameter Estimation

Parameter estimation of the model: cccDNA is estimated to have a 50-day half-life, so $\mu = \ln(2)/50$ day^{-1} [61]. Since the median rate of transcription elongation by RNA polymerase II is around 1.5 kb/min [62], we calculated the production rates $(c_1, c_2, ..., c_5)$ of all mRNAs based on their lengths to two decimal places [63, 16, 64]. These parameters are calculated based on the median value, and we checked that changes in mRNA production rate do not affect our results qualitatively. The translation rate by the ribosome is around five amino acids per second [65, 66], and we used the same approach to estimate the translation rates $(e_1, e_2, ..., e_7)$. The half-life of X protein is approximately two hours [67]. However, Kim et al. [68] have shown that expressing HBc (C protein) reduces the half-life of X protein almost by half ([68], Figure 2). Based on this observation, we estimated the half-life of X protein as one hour; thus, $\delta_x = \ln(2)$ hour^{-1}. The half-life of polymerase is also one hour [69], and we assume that the half-lives of the other proteins are the same. Cook et al. have presented a stochastic model of gene expression [70], where the average time required for gene activation is one-fourth of the product lifetime, and the activation and deactivation rates are equal. We have used the same assumption. As the half-lives of proteins are considered to be one hour, $\lambda_{off} = \lambda_{on} = 2.77$ hour^{-1}. Decay rates of mRNAs are calculated using their reported half-lives [71, 72, 73]. Since the maturation rate of pgNC to rcNC within an infected cell should be similar to the removal rate of rcNC (decay rate of rcNC μ_R and budding rate t) [74, 75], the reverse transcription rate (s_1) is equal to $\mu_R + t$ and $\mu_R = t = \ln(2)/24$ hour^{-1} [76, 5, 77].

The level of complete virions found in the blood of infected patients is up to $10^9 - 10^{10}$/ml in 8 to 10 weeks [14, 78]. The number of hepatocytes is taken to be 13.6×10^6 cells per mL [41]. As the average fraction of infected cells at the peak of infection is 97%, we can say that roughly every infected cell produces around 100–200 complete virions. Ciupe et al. [78] presented an intercellular model for HBV infection where we can observe a 10–20 day delay in the creation of productively infected cells, i.e., the phase in which they can produce complete virion particles. They also showed that the dynamic of free viruses starts with a 5–10 day delay after infection [78]. We also know that SVPs and empty virions are typically found at 1000–100,000-fold and 100-fold higher levels than complete virions, respectively, and RNA-containing particles are 100–1000-fold lower than complete virions. Based on these facts, the other parameters are chosen in a way to see these dynamics (Table S1), which has been shown in Figure 2c–f. For example, we assume that the budding rate of RNA-containing particles is 1000 times smaller than that of complete virions ($t_1 = t/1000$). Although the yield of empty virions is 100-fold higher than that of complete virions (note that their assembly is faster because they do not need to encapsidate a viral genome), we assume that they have the same budding rate as complete virions. As SVP yield is approximately

100-fold higher than that of empty virions, and since, similarly to empty virions, they do not need to encapsidate a genome, we assume that their budding rate is 100 times higher than that of empty virions ($t_2 = 100t$). Note that these are our approximations, which are consistent with *in vivo* observations [14].

As shown in Figure S3, choosing the initial number of free ribosomes equal to 10,000 provides a more realistic intracellular dynamic, and this value is a reasonable choice based on the intracellular hepatitis C virus model presented by Aunins et al. [79]. Moreover, one can observe that the number RNA Pol II does not have an impact on the dynamic of the model (Figure S3).

Parameter estimation of drugs: It has been demonstrated that various type I IFNs (IFN-α and IFN-β) bind to IFNAR2 with dissociation constant ($K_d = \frac{I_{unbind}}{I_{bind}}$) values mostly in the range 0.1–1000 nM and bind to IFNAR1 with a dissociation constant mostly in the range of 0.05–10 µM [80, 81]. In this model, we have used the smallest possible value, which is 0.1 nM. The efficacy of this drug in degrading of the cccDNA (ψ) has not been measured; therefore, in this model, we are assuming that $\psi = 0.2$. Geldanamycin is an Hsp90 inhibitor with $K_d = \frac{k_{unbind}}{k_{bind}} = 1.2$ µM [82]. Tenofovir, which is a newly developed NA, shows a greater HBV DNA suppression compared with the other NAs [83, 84]. The affinity of tenofovir is scored as the total ligand–receptor interaction energy (in Kcal/mol) and is equal to $\Delta G = -11.54$ Kcal/mol. The following equation relates the free energy of binding (ΔG) to K_d:

$$K_d = e^{\beta \Delta G},$$

where $\beta = \frac{1}{k_B T}$, k_B is the Boltzmann constant, and T is the temperature in Kelvin [85]. Therefore, for tenofovir, $K_d = 7.4$ nM. JNJ-827 is a newly developed CAM with half-maximal effective concentration of 4.7 nM. In this model, we consider the dissociation constant of this drug to also be equal to 4.7 nM.

It has been observed that increasing the binding rates of a drug does not have a significant effect on the outcome of the treatment, perhaps because this will also increase the unbinding rate, as the unbinding over binding rate is constant (dissociation constant). Thus, we assume that the binding rate of IFN is equal to b (binding rate of RNA Pol II for pgRNA), and the binding rate of NA to polymerase is equal to g (binding rate of polymerase and pgRNA). The binding rate of siRNA molecules to the pgRNA is assumed to be equal to d (binding rate of ribosome to pgRNA) with $K_d = 0.1$ nM, and the cleaving rate $k_{cleave} = 10$ hour^{-1} [86]. We also consider the PS-targeting drugs and CAMs to have the same binding rate as C protein to PSs and $k_{dis} = 10 \times \ln(2)/24 = 0.29$, which means that the half-life of rcNC:CAM is 2.4 hours (Figure S8b). In addition, we assume that the binding rate of C:CAMs is f times faster than the binding rate of C proteins for the production of an empty virion, i.e., $\kappa_{nc} = f\kappa_n$ and $\kappa_1 = f\kappa$. We assume that $f = 10$. However, changing 10 to a smaller or bigger value does not have a significant effect on the outcome of CAMs (Figure S8a). For the inhibitor of HBsAg release, we assume that its efficacy is equal to 95%.

S4 Tables and Figures

Table S1: Table of parameter values.

Parameter	Description	Parameter value	Source
μ	Removal rate of cccDNA and rcDNA	$\ln(2)/1200$ hour^{-1}	[61](*in vitro*), [5](Modelling, p.93)
λ_{off}	Silencing rate of cccDNA	2.77 hour^{-1}	[70](Modelling), cf. lines 332-336
λ_{on}	De-silencing rate of cccDNA	2.77 hour^{-1}	[70](Modelling), cf. lines 332-336
s	Impact of HBx on silencing and de-silencing of cccDNA	1/150 molecule^{-1}	Sens, Fig. S5
b	Binding rate of RNA Pol II for mRNAs	2 molecule^{-1} hour^{-1}	Sens, Fig. S5
n_{Ld}	Rate modifying for RNA Pol II binding	15	Sens, Fig. S4
c_1	Production rate of pgRNA	25.71 hour^{-1}	[62, 2], cf. lines 322-326
c_2	Production rate of LS mRNA	37.5 hour^{-1}	[62, 2], cf. lines 322-326
c_3	Production rate of S mRNA	42.86 hour^{-1}	[62, 2], cf. lines 322-326
c_4	Production rate of X mRNA	128.57 hour^{-1}	[62, 2], cf. lines 322-326
c_5	Production rate of PreC mRNA	25.71 hour^{-1}	[62, 2], cf. lines 322-326
d	Binding rate of ribosome to pgRNA	40 molecule^{-1} hour^{-1}	Sens, Fig. 2
n_{Lm}	Rate modifying for ribosome binding	25	Sens, Fig. S9
n_c	Rate modifying for ribosome binding	50	Sens, Fig. S9
n_s	Rate modifying for ribosome binding	20	Sens, Fig. S9
e_1	Elongation rate of pg:Rib_1	22.5 hour^{-1}	[65], cf. lines 326-328
e_2	Elongation rate of pg:Rib_2	49.18 hour^{-1}	[65], cf. lines 326-328
e_3	Elongation rate of LS:Rib	22.5 hour^{-1}	[65], cf. lines 326-328
e_4	Elongation rate of S:Rib_1	32.03 hour^{-1}	[65], cf. lines 326-328
e_5	Elongation rate of S:Rib_2	39.82 hour^{-1}	[65], cf. lines 326-328
e_6	Elongation rate of X:Rib	116.88 hour^{-1}	[65], cf. lines 326-328
e_7	Elongation rate of PreC:Rib	84.91 hour^{-1}	[65], cf. lines 326-328
d_m	Degradation rate of pgRNA	$\ln(2)/5$ hour^{-1}	[71](*in vivo*, Huh-7, Fig. 4)
d_l	Degradation rate of LS mRNA	$\ln(2)/3$ hour^{-1}	[72](*in vivo*, HepG2, Fig. 7A*)
d_s	Degradation rate of S mRNA	$\ln(2)/3$ hour^{-1}	[72](*in vivo*, HepG2, Fig. 7A*)
d_x	Degradation rate of X mRNA	$\ln(2)$ hour^{-1}	[73](*in vivo*, HepG2, Fig. 8)
d_p	Degradation rate of PreC mRNA	$\ln(2)/5$ hour^{-1}	[72](*in vivo*, HepG2, Fig. 4)
δ_p	Degradation rate of polymerase	$\ln(2)$ hour^{-1}	[69](*in vivo*, HepG2, Fig. 2B)
δ_c	Degradation rate of C protein	$\ln(2)$ hour^{-1}	Assumed=δ_p, δ_x
δ_l	Degradation rate of L protein	$\ln(2)$ hour^{-1}	Assumed=δ_p, δ_x
δ_m	Degradation rate of M protein	$\ln(2)$ hour^{-1}	Assumed=δ_p, δ_x
δ_s	Degradation rate of S protein	$\ln(2)$ hour^{-1}	Assumed=δ_p, δ_x
δ_x	Degradation rate of X protein	$\ln(2)$ hour^{-1}	[68, 67], cf. lines 328-331
δ_{pc}	Degradation rate of PreC protein	$\ln(2)$ hour^{-1}	Assumed=δ_p, δ_x
g	Binding rate of P and pgRNA	5 molecule^{-1} hour^{-1}	Sens, Fig. S5
$K_{d_{PS}}$	dissociation constant of C binding to PSs	4 nM	[87, 22](*in vitro*)
$\kappa_{n_{PS}}$	Binding rate of C to PSs	24 molecule^{-1} hour^{-1}	Sens, Fig. S9
κ	Forward rate for elongation reactions	$60,000$ molecule^{-1} hour^{-1}	Assumed=κ_1
s_1	mature nucleocapsid production rate	$\ln(2)/12$ hour^{-1}	[74, 75, 5](Modelling), cf. lines 337-340
t	Virion budding rate	0.029 hour^{-1}	[76](*in vivo***), [5](Modelling)
t_1	RNA-containing particle budding rate	0.000029 hour^{-1}	[14], cf. lines 352-360
t_2	Spherical SVPs budding rate	2.9 hour^{-1}	[14], cf. lines 352-360
t_3	Filamentous SVPs budding rate	8.7 hour^{-1}	Sens, Fig. S9
κ_1	Binding rate of S proteins	$60,000$ hour^{-1}	Sens***
λ	The impact of the L protein on particles release rate	3×10^{-4} molecule^{-1}	Sens, Fig. S4
μ_R	Removal rate of capsids	$\ln(2)/24$ hour^{-1}	[77](*in vitro*, cell line AML12, Fig. 4C)
κ_n	Forward rate for nucleation reactions	600 molecule^{-1} hour^{-1}	Sens, Fig. S9
nuc	Number of core proteins composing the nucleating structure	3	[24](*in vitro* and Modelling)
RNA Pol	Number of RNA Pol II	150 molecules	Sens, Fig. S3
Rib	Number of ribosomes	10,000 molecules	Sens, Fig. S3

Sens: Sensitivity analysis was performed.

* These values are approximated based on Figure 7A in [72].

** Measured in an animal system.

*** Sensitivity analysis was performed, and this value was chosen to get SVPs at a 10,000-fold higher level than complete virions.

Table S2: Table of dissociation constants of PS-targeting compounds

Compound No	K_d for PS1	K_d for PS2	K_d for PS3
15	32.2355875806	6.770135567	1.152453487
20	11.9307880554	66012.7052868772	2286.7843145983
21	0.5510732927	30379.610743961	3934.1801166796
24	15.8322201448	14364.0590410873	44.6878235885
26	66.1920556277	2764.7217021614	52.0446956707
28	883.7449892914	57.5417086996	677.7661477891

All the rates are in nM and are measured *in vitro*.

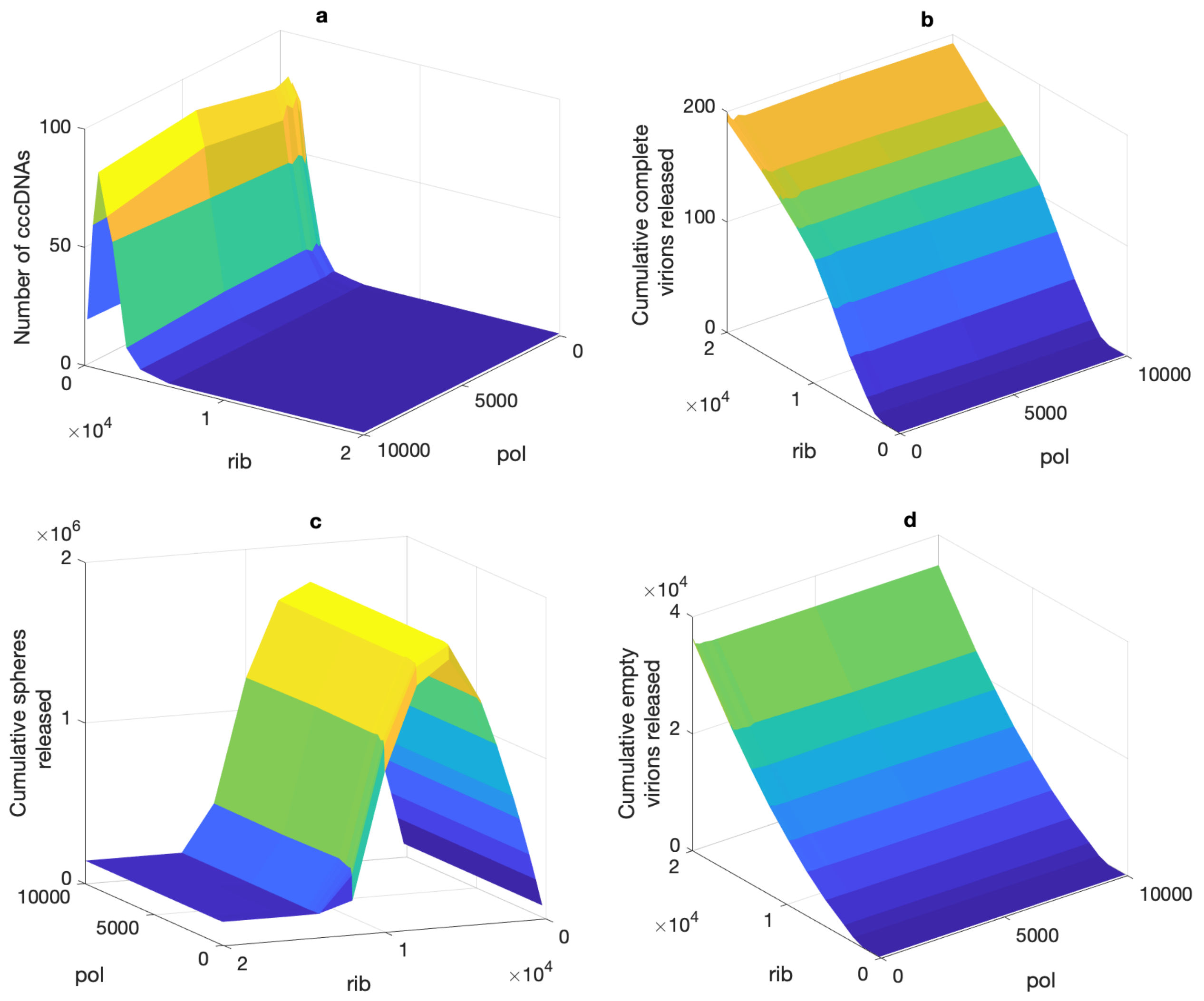

Figure S3: The number of ribosomes (rib) plays an important role in the dynamics of the model, while the amount of RNA Pol II (pol) in the nucleus does not have any impact. This figure shows the number of cccDNAs and total released particles per cell with parameter values from Table S1. (a) Number of cccDNAs. (b–d) Total complete virions, spherical SVPs, and empty virions released, respectively.

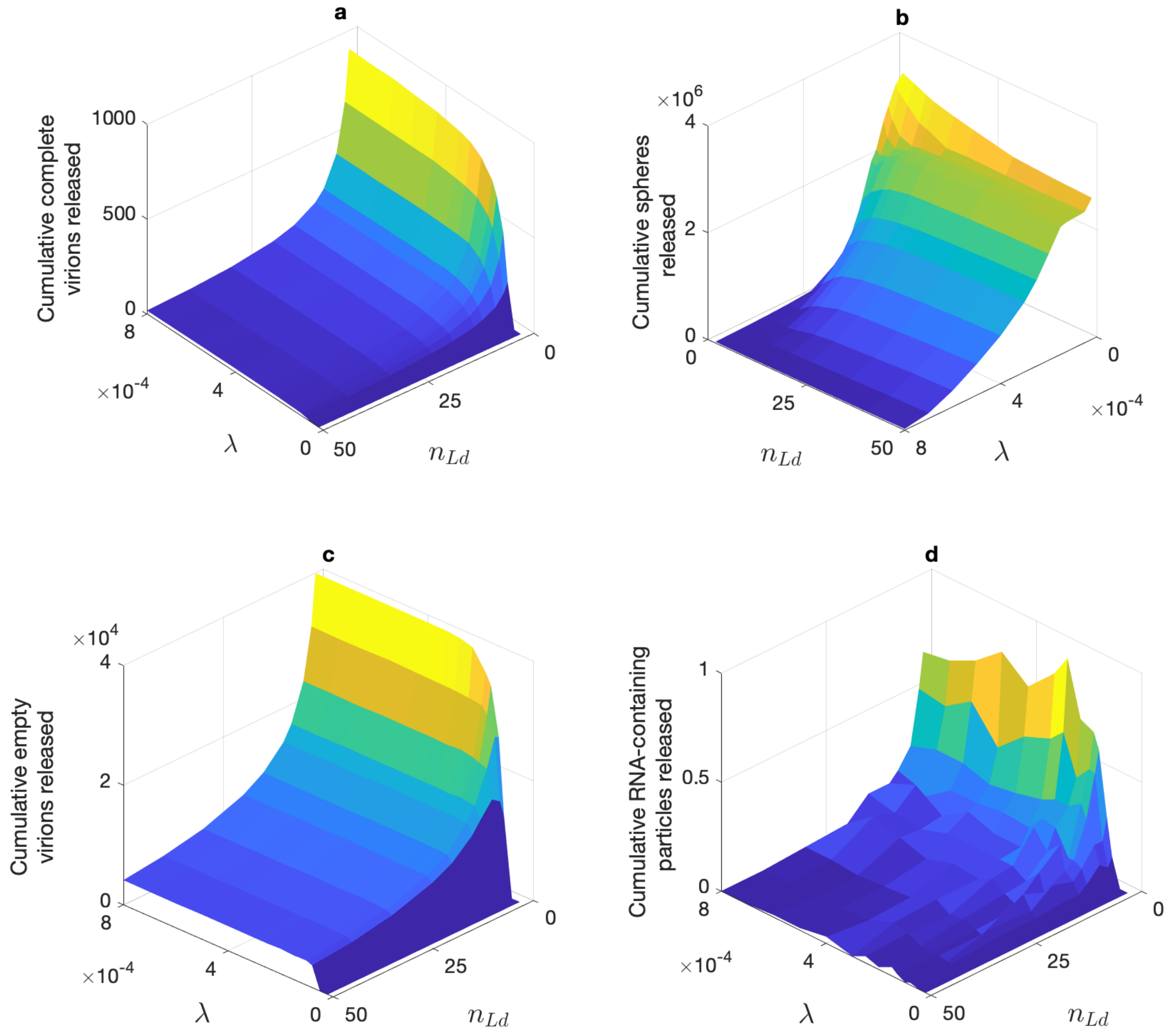

Figure S4: If the parameter λ has a biologically relevant value, i.e., the number of cccDNAs does not increase rapidly ($\lambda > 2 \times 10^{-4}$), this parameter does not have any impact on released particles except spheres, while n_{Ld} shows an opposite effect. This figure shows total released particles per cell with parameter values from Table S1. (a) Total released complete virions. (b–d) Total spherical SVPs, empty virions, and RNA-containing particles released, respectively.

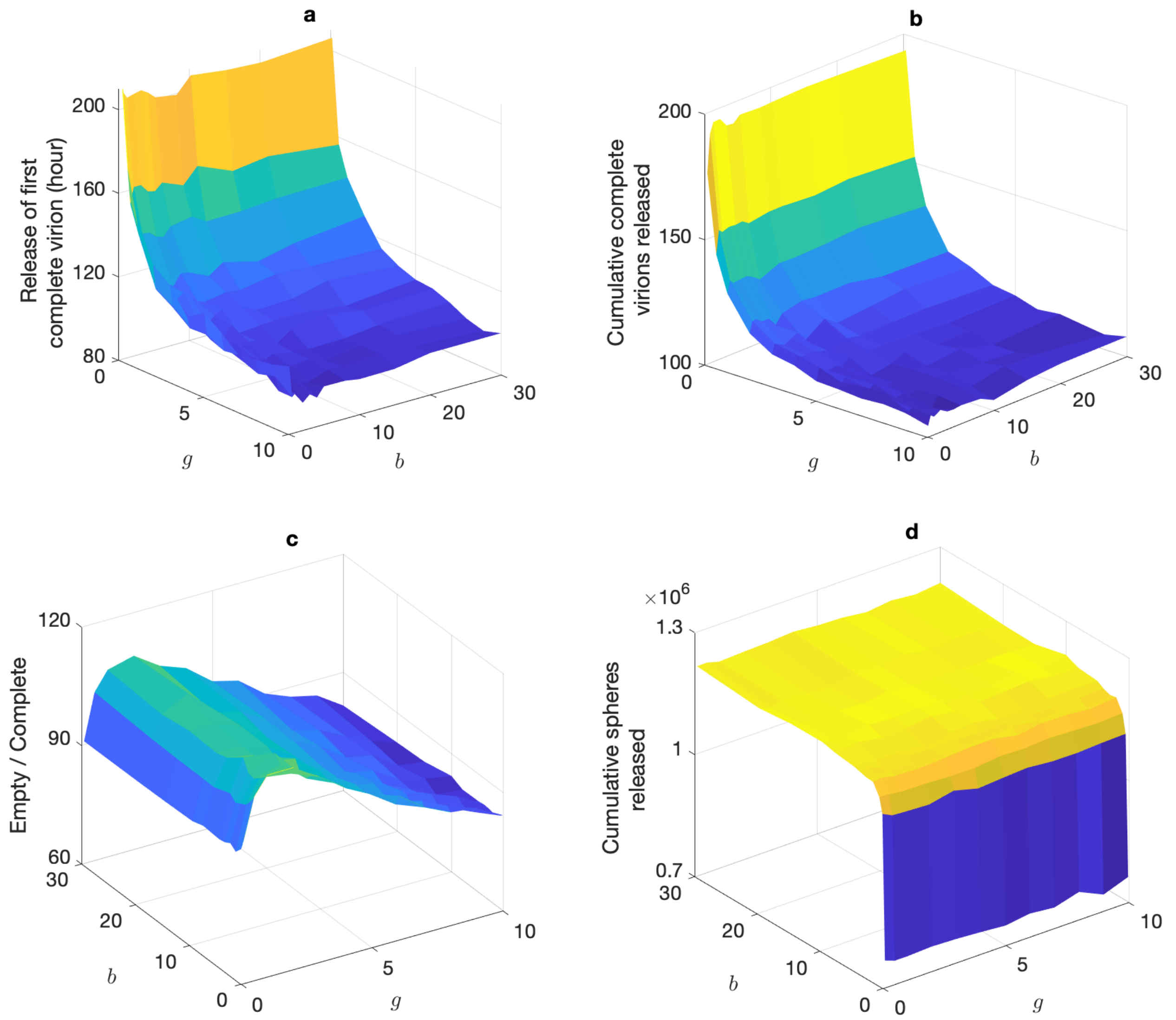

Figure S5: The binding rate of RNA Pol II to cccDNA (Step 3; Figure S13) does not have any impacts on the dynamics of the model, while the binding rate of P to the pgRNA (Step 5; Figure S13) plays an important role. This figure shows the dynamic of the model with parameter values from Table S1. (a) The time that the first complete virion is released after infection. (b,d) Total complete virions and spherical SVPs released, respectively. (c) The ratio of total empty virions released over complete virions. The parameter s (impact of HBx on silencing and de-silencing of cccDNA) indicates the same behaviour as b, i.e., it does not have any impacts on the dynamics of the model.

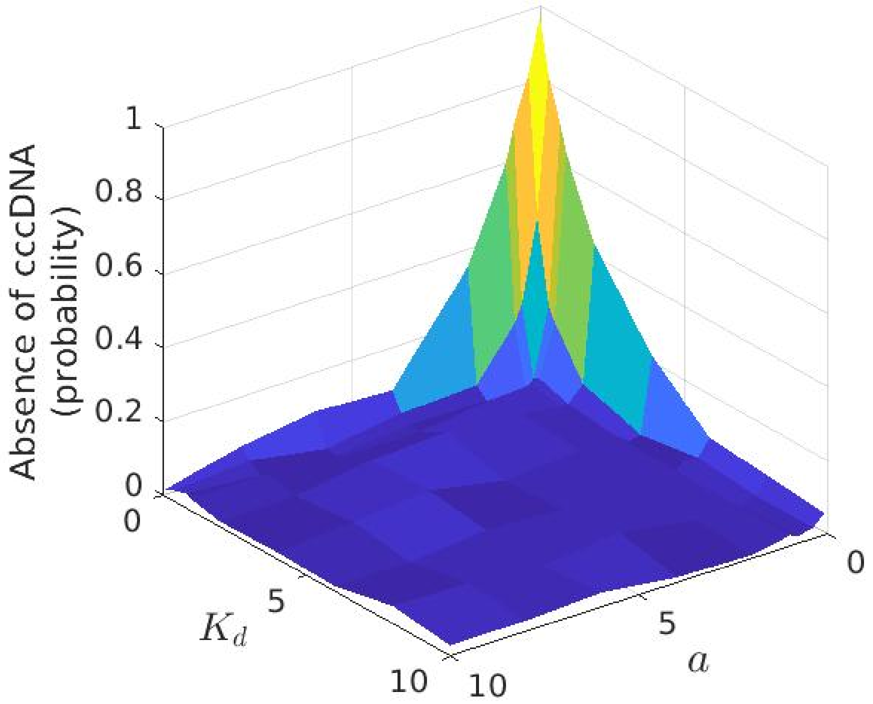

Figure S6: The probability of prevention of cccDNA formation with anti-host DNA repair factors as a function of the parameter a and the binding affinity (K_d) of this drug, with parameter values from Table S1 and $\alpha = 1$ hour^{-1}.

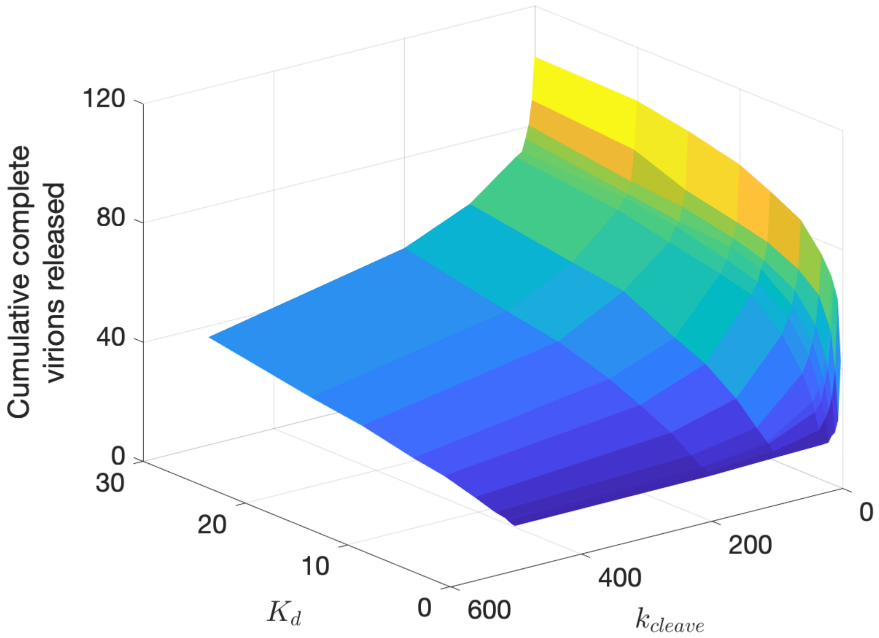

Figure S7: The number of complete virions released with RNA interference treatment and parameter values from Table S1 as a function of the binding affinity (K_d) and the cleaving rate (k_{cleave}) of this drug. The concentration of the drug is 50 molecules.

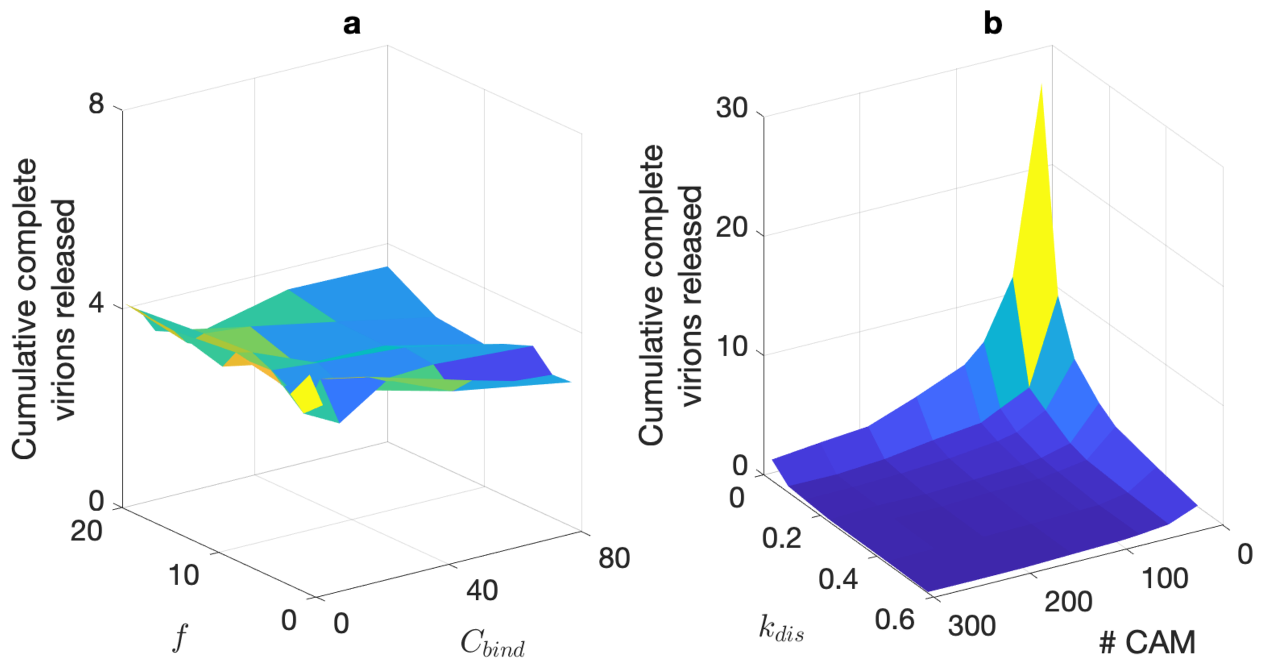

Figure S8: The number of complete virions released with CAM treatment and parameter values from Table S1. In (a), $k_{dis} = 0.29$ and concentration of CAM is 20 molecules. In (b), $f = 10$ and $C_{bind} = 24$.

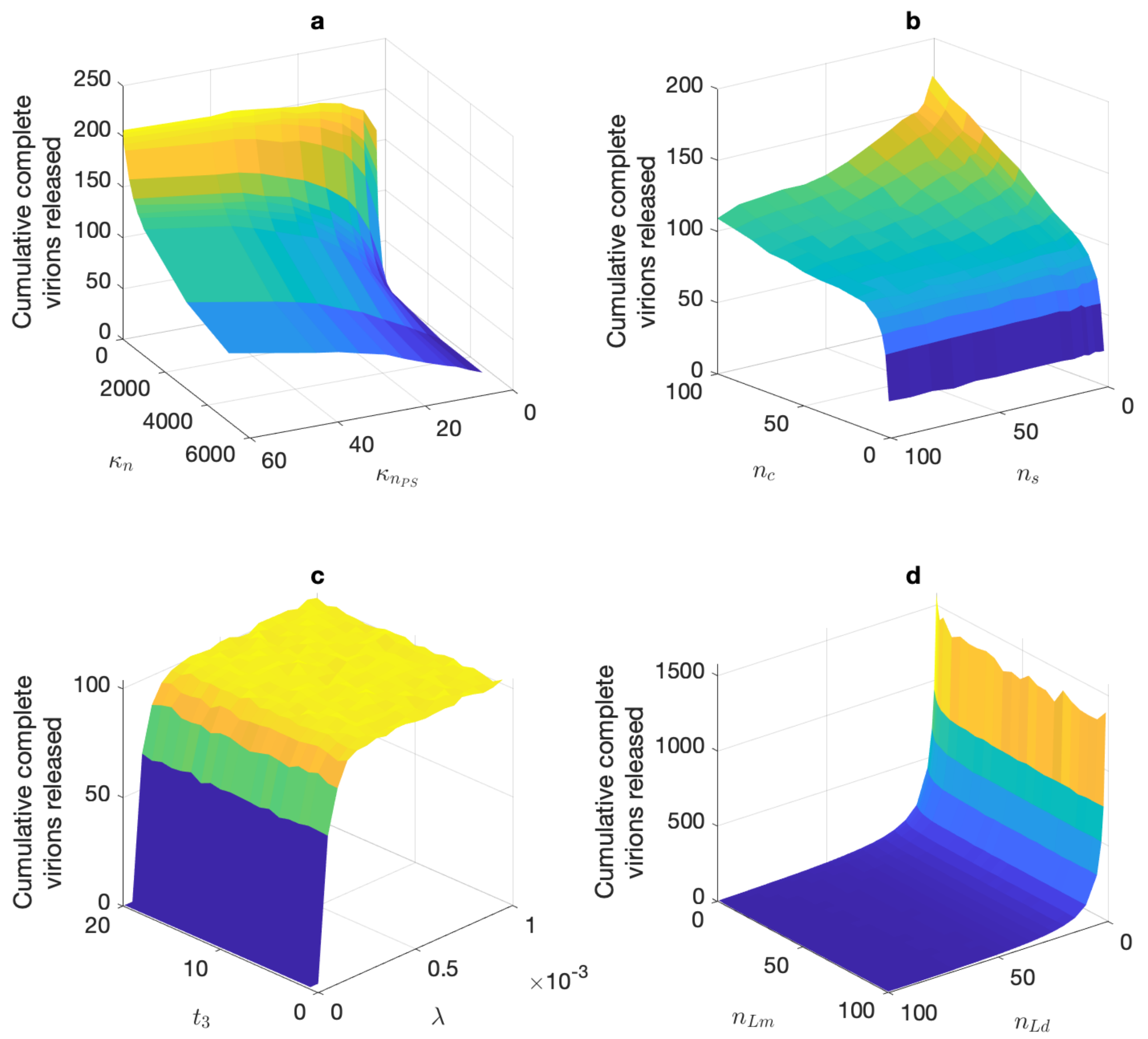

Figure S9: The number of complete virions released with parameter values from Table S1.

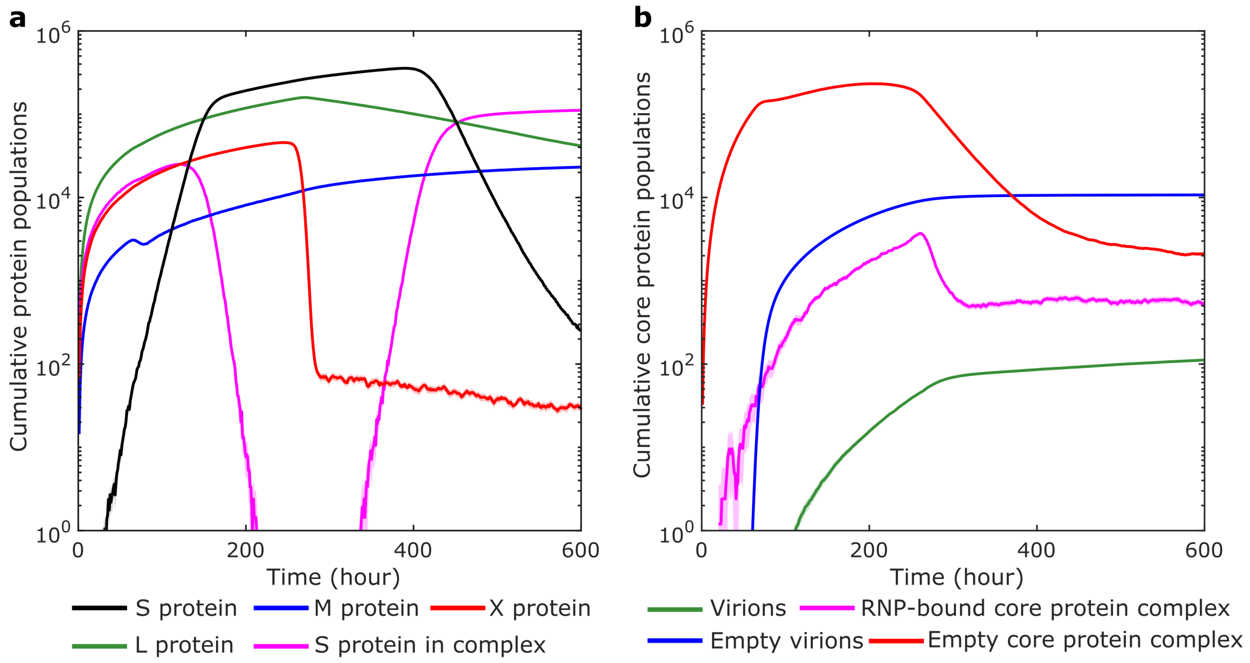

Figure S10: The average of cumulative populations of proteins inside the cell over 100 simulations. Shaded areas around curves indicate the 95% CI.

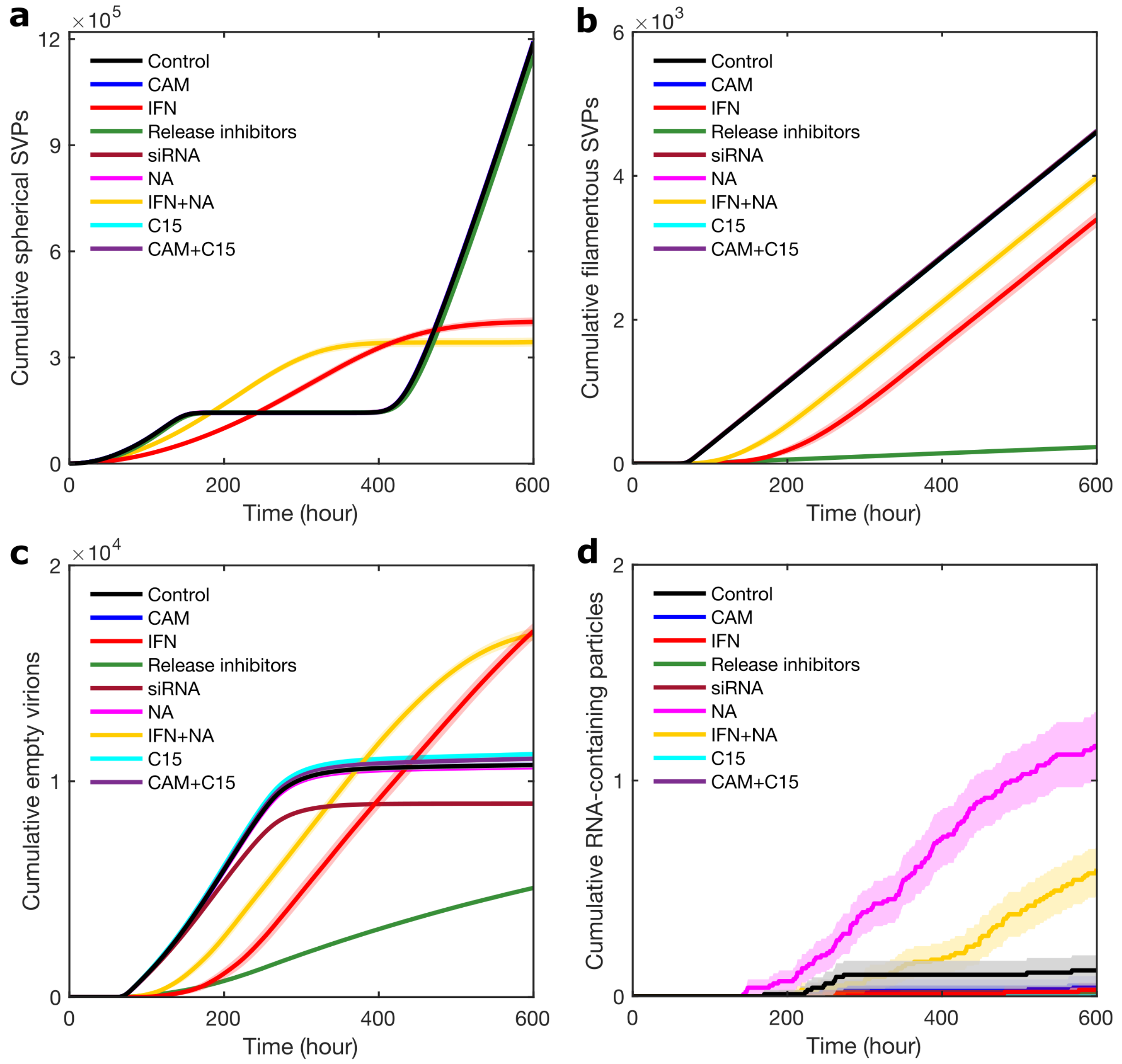

Figure S11: The dynamics of incomplete particles released with different treatment options after cccDNA formation, with treatment starting with the initial condition cccDNA = 1. A free concentration of 20 molecules for drugs is considered, except for NA, which is equal to 100; for IFN+NA, we consider IFN = 10 and NA = 50 (half concentration for each drug), and similarly, for CAM+C15, we consider CAM = 10 and C15 = 10. Black shows the absence of treatment (Control) for comparison. (a) and (b) show the dynamics of spherical and filamentous SVPs, respectively. (c) and (d) indicate the dynamics of empty virions and RNA-containing particles, respectively.

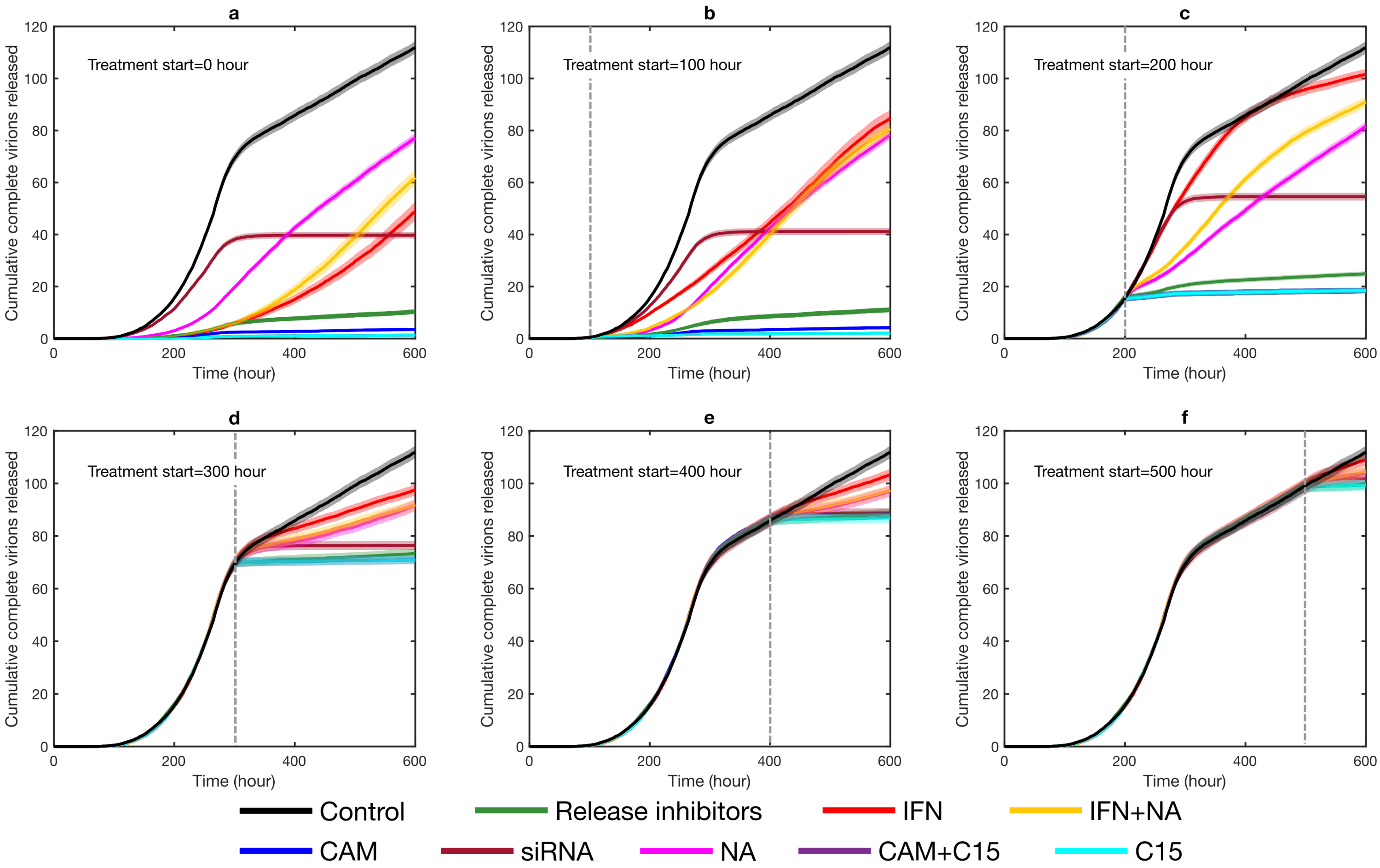

Figure S12: The number of complete virions released with starting treatment at different times post-infection and parameter values from Table S1. The grey dashed line shows the start of treatment and the shaded area around each curve indicates the 95% CI.

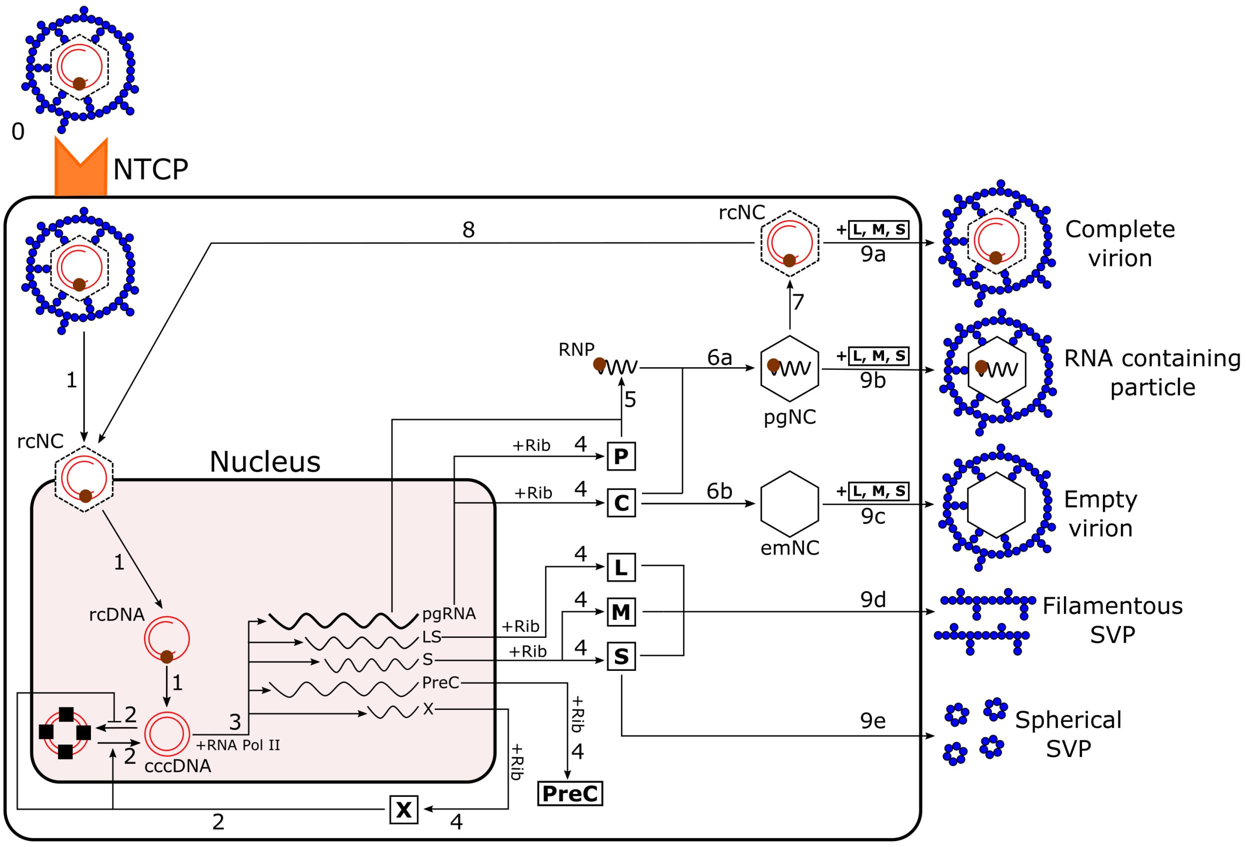

Figure S13: The HBV life cycle. Step 0: Viral entry is mediated by the sodium-taurocholate co-transporting polypeptide (NTCP) receptor, which allows the nucleocapsid (rcNC) containing relaxed circular DNA (rcDNA) to be released into the cell via endocytosis. This step is not included explicitly in the model. Step 1: After attachment of the rcNC to the nucleus, it delivers the rcDNA. The host DNA repair factors convert the rcDNA into covalently closed circular DNA (cccDNA). Step 2: X protein, which gets produced by the cccDNA over the course of infection, promotes the de-silencing of the cccDNA and blocks its silencing. Step 3: The cccDNA is used as a template by RNA Polymerase II (RNA Pol II) to synthesise viral RNAs, including the pgRNA; LS, S, PreC, and X mRNAs. The pgRNA encodes both the core (C) and polymerase (P) proteins; X mRNA the X protein; LS mRNA the L surface protein; S mRNA the M and S surface proteins; and PreC mRNA the precore (PreC) protein. Step 4: Translation of these mRNAs by ribosomes (Rib) leading to synthesis of viral proteins. Step 5: The pgRNA and P form a 1:1 ribonucleoprotein (RNP) complex, which is assembly competent. Step 6a: Encapsidation of the RNP complex by C proteins to form a nucleocapsid containing pgRNA:P (pgNC), also termed the immature nucleocapsid. Step 6b: Assembly of C proteins into an empty nucleocapsid (emNC). Step 7: The reverse transcription of pgRNA by P within the pgNC, resulting in the conversion of pgNC into a rcDNA containing nucleocapsid (rcNC), which is also termed the mature nucleocapsid. Step 8: Recycling of the rcDNA from mature nucleocapsids to form more cccDNAs. Step 9a: Envelopment of mature nucleocapsids within a membrane layer containing the surface proteins L, M, and S, leading to the secretion of a complete virion. Step 9b: Envelopment of an immature nucleocapsid resulting in secretion of an RNA-containing particle. Step 9c: Envelopment of an empty nucleocapsid and secretion of an empty virion. Step 9d: L, M, and S proteins form empty filaments and filamentous subviral particles (SVP), via conventional tubular budding, into late endosomes and exit the cell. Step 9e: S proteins assemble into octahedral spheres (spherical SVPs) and exit the cell.

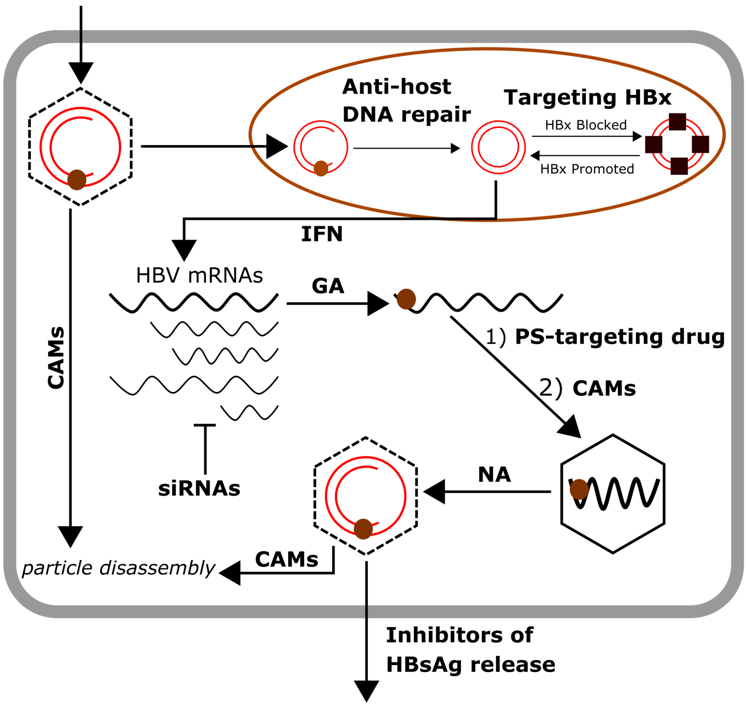

Figure S14: Current and potential future drugs for the treatment of HBV. The different types of drugs are labelled next to the viral life cycle steps they target. CAMs both inhibit nucleocapsid assembly and promote disassembly of mature nucleocapsids, either following entry or prior to release, and are therefore indicated at multiple steps of the viral life cycle. Interferon (IFN)-based therapy blocks the transcription step. siRNAs, which reduce mRNA levels, are indicated by the bar-headed line. Geldanamycin (GA) prevents the formation of the RNP complex. Nucleot(s)ide analogues (NAs) inhibit the progression of immature into mature nucleocapsids. PS-targeting drugs bind to the PSs of HBV and inhibit nucleocapsid assembly. Inhibitors of HBsAg release target viral and SVP egress.

References

[1] Yan, H.; Zhong, G.; Xu, G.; He, W.; Jing, Z.; Gao, Z.; Huang, Y.; Qi, Y.; Peng, B.; Wang, H.; et al.. Sodium taurocholate cotransporting polypeptide is a functional receptor for human hepatitis B and D virus. *elife* **2012**, *1*, e00049.

[2] Hu, J. Hepatitis B virus virology and replication. In *Hepatitis B virus in human diseases*; Springer, 2016; pp. 1–34.

[3] Hu, J.; Seeger, C. Hepadnavirus genome replication and persistence. *Cold Spring Harb. Perspect. Med.* **2015**, *5*, a021386.

[4] Hanawalt, P.C.; Spivak, G. Transcription-coupled DNA repair: two decades of progress and surprises. *Nat. Rev. Mol. cell Biol.* **2008**, *9*, 958.

[5] Murray, J.M.; Goyal, A. In silico single cell dynamics of hepatitis B virus infection and clearance. *J. Theor. Biol.* **2015**, *366*, 91–102.

[6] Newbold, J.E.; Xin, H.; Tencza, M.; Sherman, G.; Dean, J.; Bowden, S.; Locarnini, S. The covalently closed duplex form of the hepadnavirus genome exists in situ as a heterogeneous population of viral minichromosomes. *J. Virol.* **1995**, *69*, 3350–3357.

[7] Shi, L.; Li, S.; Shen, F.; Li, H.; Qian, S.; Lee, D.H.S.; Wu, J.Z.; Yang, W. Characterization of nucleosome positioning in hepadnaviral covalently closed circular DNA minichromosomes. *J. Virol.* **2012**, *86*, 10059–10069.

[8] Belloni, L.; Pollicino, T.; De Nicola, F.; Guerrieri, F.; Raffa, G.; Fanciulli, M.; Raimondo, G.; Levrero, M. Nuclear HBx binds the HBV minichromosome and modifies the epigenetic regulation of cccDNA function. *Proc. Natl. Acad. Sci. USA* **2009**, *106*, 19975–19979.

[9] Belloni, L.; Allweiss, L.; Guerrieri, F.; Pediconi, N.; Volz, T.; Pollicino, T.; Petersen, J.; Raimondo, G.; Dandri, M.; Levrero, M. IFN-α inhibits HBV transcription and replication in cell culture and in humanized mice by targeting the epigenetic regulation of the nuclear cccDNA minichromosome. *J. Clin. Invest.* **2012**, *122*, 529–537.

[10] Liu, F.; Campagna, M.; Qi, Y.; Zhao, X.; Guo, F.; Xu, C.; Li, S.; Li, W.; Block, T.M.; Chang, J.; et al.. Alpha-interferon suppresses hepadnavirus transcription by altering epigenetic modification of cccDNA minichromosomes. *PLoS Pathog.* **2013**, *9*, e1003613.

[11] Lucifora, J.; Arzberger, S.; Durantel, D.; Belloni, L.; Strubin, M.; Levrero, M.; Zoulim, F.; Hantz, O.; Protzer, U. Hepatitis B virus X protein is essential to initiate and maintain virus replication after infection. *J. Hepatol.* **2011**, *55*, 996–1003.

[12] Nassal, M. HBV cccDNA: viral persistence reservoir and key obstacle for a cure of chronic hepatitis B. *Gut* **2015**, *64*, 1972–1984.

[13] Jansen, M.; Pfaffelhuber, P. Stochastic gene expression with delay. *J. Theor. Biol.* **2015**, *364*, 355–363.

[14] Hu, J.; Liu, K. Complete and incomplete hepatitis B virus particles: formation, function, and application. *Viruses* **2017**, *9*, 56.

[15] Zhu, R.; Salahub, D. Delay stochastic simulation of single-gene expression reveals a detailed relationship between protein noise and mean abundance. *FEBS Lett.* **2008**, *582*, 2905–2910.

[16] Prange, R. Host factors involved in hepatitis B virus maturation, assembly, and egress. *Med. Microbiol. Immunol.* **2012**, *201*, 449–461.

[17] Hu, J.; Toft, D.O.; Seeger, C. Hepadnavirus assembly and reverse transcription require a multi-component chaperone complex which is incorporated into nucleocapsids. *EMBO J.* **1997**, *16*, 59–68.

[18] Junker-Niepmann, M.; Bartenschlager, R.; Schaller, H. A short cis-acting sequence is required for hepatitis B virus pregenome encapsidation and sufficient for packaging of foreign RNA. *EMBO J.* **1990**, *9*, 3389–3396.

[19] Hirsch, R.C.; Loeb, D.D.; Pollack, J.R.; Ganem, D. cis-acting sequences required for encapsidation of duck hepatitis B virus pregenomic RNA. *J. Virol.* **1991**, *65*, 3309–3316.

[20] Bartenschlager, R.; Schaller, H. Hepadnaviral assembly is initiated by polymerase binding to the encapsidation signal in the viral RNA genome. *EMBO J.* **1992**, *11*, 3413–3420.

[21] Hu, J.; Lin, L. RNA-protein interactions in hepadnavirus reverse transcription. *Front. Biosci.* **2009**, *14*, 1606.

[22] Patel, N.; White, S.J.; Thompson, R.F.; Bingham, R.; Weiß, E.U.; Maskell, D.P.; Zlotnick, A.; Dykeman, E.C.; Tuma, R.; Twarock, R.; et al.. HBV RNA pre-genome encodes specific motifs that mediate interactions with the viral core protein that promote nucleocapsid assembly. *Nat. Microbiol.* **2017**, *2*, 17098.

[23] Endres, D.; Zlotnick, A. Model-based analysis of assembly kinetics for virus capsids or other spherical polymers. *Biophys. J.* **2002**, *83*, 1217–1230.

[24] Porterfield, J.Z.; Zlotnick, A. An overview of capsid assembly kinetics. In *Emerging Topics in Physical Virology*; Imperial College Press, 2010; pp. 131–158.

[25] Tu, T.; Budzinska, M.; Shackel, N.; Urban, S. HBV DNA integration: molecular mechanisms and clinical implications. *Viruses* **2017**, *9*, 75.

[26] Summers, J.; Smith, P.M.; Horwich, A.L. Hepadnavirus envelope proteins regulate covalently closed circular DNA amplification. *J. Virol.* **1990**, *64*, 2819–2824.

[27] Schreiner, S.; Nassal, M. A role for the host DNA damage response in hepatitis B virus cccDNA formation—and beyond? *Viruses* **2017**, *9*, 125.

[28] Nishi, R.; Wijnhoven, P.W.G.; Kimura, Y.; Matsui, M.; Konietzny, R.; Wu, Q.; Nakamura, K.; Blundell, T.L.; Kessler, B.M. The deubiquitylating enzyme UCHL3 regulates Ku80 retention at sites of DNA damage. *Sci. Rep.* **2018**, *8*, 17891.

[29] Lambert, C.; Döring, T.; Prange, R. Hepatitis B virus maturation is sensitive to functional inhibition of ESCRT-III, Vps4, and γ2-adaptin. *J. Virol.* **2007**, *81*, 9050–9060.

[30] Jiang, B.; Himmelsbach, K.; Ren, H.; Boller, K.; Hildt, E. Subviral hepatitis B virus filaments, like infectious viral particles, are released via multivesicular bodies. *J. Virol.* **2016**, *90*, 3330–3341.

[31] Patient, R.; Hourioux, C.; Roingeard, P. Morphogenesis of hepatitis B virus and its subviral envelope particles. *Cell. Microbiol.* **2009**, *11*, 1561–1570.

[32] Gilbert, R.J.C.; Beales, L.; Blond, D.; Simon, M.N.; Lin, B.Y.; Chisari, F.V.; Stuart, D.I.; Rowlands, D.J. Hepatitis B small surface antigen particles are octahedral. *Proc. Natl. Acad. Sci. USA* **2005**, *102*, 14783–14788.

[33] Dryden, K.A.; Wieland, S.F.; Whitten-Bauer, C.; Gerin, J.L.; Chisari, F.V.; Yeager, M. Native hepatitis B virions and capsids visualized by electron cryomicroscopy. *Mol. Cell* **2006**, *22*, 843–850.

[34] Short, J.M.; Chen, S.; Roseman, A.M.; Butler, P.J.G.; Crowther, R.A. Structure of hepatitis B surface antigen from subviral tubes determined by electron cryomicroscopy. *J. Mol. Biol.* **2009**, *390*, 135–141.

[35] Revill, P.A.; Chisari, F.V.; Block, J.M.; Dandri, M.; Gehring, A.J.; Guo, H.; Hu, J.; Kramvis, A.; Lampertico, P.; Janssen, H.L.A.; et al.. A global scientific strategy to cure hepatitis B. *Lancet Gastroenterol. Hepatol.* **2019**.

[36] Wang, X.Y.; Wei, Z.M.; Wu, G.Y.; Wang, J.H.; Zhang, Y.J.; Li, J.; Zhang, H.H.; Xie, X.W.; Wang, X.; Wang, Z.H.; et al.. In vitro inhibition of HBV replication by a novel compound, GLS4, and its efficacy against adefovir-dipivoxil-resistant HBV mutations. *Antivir. Ther.* **2012**, *17*, 793–803.

[37] van Bömmel, F.; Bartens, A.; Mysickova, A.; Hofmann, J.; Krüger, D.H.; Berg, T.; Edelmann, A. Serum hepatitis B virus RNA levels as an early predictor of hepatitis B envelope antigen seroconversion during treatment with polymerase inhibitors. *Hepatology* **2015**, *61*, 66–76.

[38] Rokuhara, A.; Matsumoto, A.; Tanaka, E.; Umemura, T.; Yoshizawa, K.; Kimura, T.; Maki, N.; Kiyosawa, K. Hepatitis B virus RNA is measurable in serum and can be a new marker for monitoring lamivudine therapy. *J. Gastroenterol.* **2006**, *41*, 785–790.

[39] Huang, Y.W.; Chayama, K.; Tsuge, M.; Takahashi, S.; Hatakeyama, T.; Abe, H.; Hu, J.T.; Liu, C.J.; Lai, M.Y.; Chen, D.S.; et al.. Differential effects of interferon and lamivudine on serum HBV RNA inhibition in patients with chronic hepatitis B. *Antivir. Ther.* **2010**, *15*, 177–184.

[40] Huang, Y.W.; Takahashi, S.; Tsuge, M.; Chen, C.L.; Wang, T.C.; Abe, H.; Hu, J.T.; Chen, D.S.; Yang, S.S.; Chayama, K.; et al.. On-treatment low serum HBV RNA level predicts initial virological response in chronic hepatitis B patients receiving nucleoside analogue therapy. *Antivir. Ther.* **2015**, *20*, 369–375.

[41] Ciupe, S.M.; Ribeiro, R.M.; Perelson, A.S. Antibody responses during hepatitis B viral infection. *PLoS Comput. Biol.* **2014**, *10*, e1003730.

[42] Roingeard, P.; Sureau, C. Ultrastructural analysis of hepatitis B virus in HepG2-transfected cells with special emphasis on subviral filament morphogenesis. *Hepatology* **1998**, *28*, 1128–1133.

[43] Patient, R.; Hourioux, C.; Sizaret, P.Y.; Trassard, S.; Sureau, C.; Roingeard, P. Hepatitis B virus subviral envelope particle morphogenesis and intracellular trafficking. *J. Virol.* **2007**, *81*, 3842–3851.

[44] Molnar-Kimber, K.L.; Jarocki-Witek, V.; Dheer, S.K.; Vernon, S.K.; Conley, A.J.; Davis, A.R.; others. Distinctive properties of the hepatitis B virus envelope proteins. *J. Virol.* **1988**, *62*, 407–416.

[45] Fatehi Chenar, F.; Kyrychko, Y.N.; Blyuss, K.B. Mathematical model of immune response to hepatitis B. *J. Theor. Biol.* **2018**, *447*, 98–110.

[46] Micco, L.; Peppa, D.; Loggi, E.; Schurich, A.; Jefferson, L.; Cursaro, C.; Panno, A.M.; Bernardi, M.; Brander, C.; Bihl, F.; et al.. Differential boosting of innate and adaptive antiviral responses during pegylated-interferon-alpha therapy of chronic hepatitis B. *J. Hepatol.* **2013**, *58*, 225–233.

[47] Lucifora, J.; Xia, Y.; Reisinger, F.; Zhang, K.; Stadler, D.; Cheng, X.; Sprinzl, M.F.; Koppensteiner, H.; Makowska, Z.; Volz, T.; et al.. Specific and nonhepatotoxic degradation of nuclear hepatitis B virus cccDNA. *Science* **2014**, *343*, 1221–1228.

[48] Thimme, R.; Dandri, M. Dissecting the divergent effects of interferon-alpha on immune cells: time to rethink combination therapy in chronic hepatitis B? *J. Hepatol.* **2013**, *58*, 205–209.

[49] Locarnini, S.; Hatzakis, A.; Chen, D.S.; Lok, A. Strategies to control hepatitis B: Public policy, epidemiology, vaccine and drugs. *J. Hepatol.* **2015**, *62*, S76–S86.

[50] Zoulim, F.; Durantel, D. Antiviral therapies and prospects for a cure of chronic hepatitis B. *Cold Spring Harb. Perspect. Med.* **2015**, *5*, a021501.

[51] Whitesell, L.; Mimnaugh, E.G.; De Costa, B.; Myers, C.E.; Neckers, L.M. Inhibition of heat shock protein HSP90-pp60v-src heteroprotein complex formation by benzoquinone ansamycins: essential role for stress proteins in oncogenic transformation. *Proc. Natl. Acad. Sci. USA* **1994**, *91*, 8324–8328.

[52] Johnson, J.L.; Toft, D.O. Binding of p23 and hsp90 during assembly with the progesterone receptor. *Mol. Endocrinol.* **1995**, *9*, 670–678.

[53] Smith, D.F.; Whitesell, L.; Nair, S.C.; Chen, S.; Prapapanich, V.; Rimerman, R.A. Progesterone receptor structure and function altered by geldanamycin, an hsp90-binding agent. *Mol. Cell. Biol.* **1995**, *15*, 6804–6812.

[54] Guo, F.; Zhao, Q.; Sheraz, M.; Cheng, J.; Qi, Y.; Su, Q.; Cuconati, A.; Wei, L.; Du, Y.; Li, W.; et al.. HBV core protein allosteric modulators differentially alter cccDNA biosynthesis from de novo infection and intracellular amplification pathways. *PLoS Pathog.* **2017**, *13*, e1006658.

[55] Zoulim, F.; Yogaratnam, J.Z.; Vandenbossche, J.J.; Lenz, O.; Talloen, W.; Vistuer, C.; Moscalu, I.; Streinu-Cercel, A.; Bourgeois, S.; Buti, M.; et al.. Safety, Tolerability, Pharmacokinetics and Antiviral Activity of JNJ-56136379, a Novel HBV Capsid Assembly Modulator, in Non-cirrhotic, Treatment-naive Subjects with Chronic Hepatitis B. Hepatology, 2017, Vol. 66, pp. 1263A–1264A.

[56] Berke, J.M.; Dehertogh, P.; Vergauwen, K.; Van Damme, E.; Mostmans, W.; Vandyck, K.; Pauwels, F. Capsid assembly modulators have a dual mechanism of action in primary human hepatocytes infected with hepatitis B virus. *Antimicrob. Agents Chemother.* **2017**, *61*, e00560–17.

[57] Schlicksup, C.J.; Wang, J.C.Y.; Francis, S.; Venkatakrishnan, B.; Turner, W.W.; VanNieuwenhze, M.; Zlotnick, A. Hepatitis B virus core protein allosteric modulators can distort and disrupt intact capsids. *Elife* **2018**, *7*, e31473.

[58] Lahlali, T.; Berke, J.M.; Vergauwen, K.; Foca, A.; Vandyck, K.; Pauwels, F.; Zoulim, F.; Durantel, D. Novel potent capsid assembly modulators regulate multiple steps of the hepatitis B virus life cycle. *Antimicrob. Agents Chemother.* **2018**, *62*, e00835–18.

[59] Billioud, G.; Pichoud, C.; Puerstinger, G.; Neyts, J.; Zoulim, F. The main hepatitis B virus (HBV) mutants resistant to nucleoside analogs are susceptible in vitro to non-nucleoside inhibitors of HBV replication. *Antiviral Res.* **2011**, *92*, 271–276.

[60] Zlotnick, A.; Mukhopadhyay, S. Virus assembly, allostery, and antivirals. *Trends Microbiol.* **2011**, *19*, 14–23.

[61] Chong, C.L.; Chen, M.L.; Wu, Y.C.; Tsai, K.N.; Huang, C.C.; Hu, C.p.; Jeng, K.S.; Chou, Y.C.; Chang, C. Dynamics of HBV cccDNA expression and transcription in different cell growth phase. *J. Biomed. Sci.* **2011**, *18*, 96.

[62] Veloso, A.; Kirkconnell, K.S.; Magnuson, B.; Biewen, B.; Paulsen, M.T.; Wilson, T.E.; Ljungman, M. Rate of elongation by RNA polymerase II is associated with specific gene features and epigenetic modifications. *Genome Res.* **2014**, *24*, 896–905.

[63] Liang, T.J. Hepatitis B: the virus and disease. *Hepatology* **2009**, *49*, S13–S21.

[64] Milich, D.; Liang, T.J. Exploring the biological basis of hepatitis B e antigen in hepatitis B virus infection. *Hepatology* **2003**, *38*, 1075–1086.

[65] Ingolia, N.T.; Lareau, L.F.; Weissman, J.S. Ribosome profiling of mouse embryonic stem cells reveals the complexity and dynamics of mammalian proteomes. *Cell* **2011**, *147*, 789–802.

[66] Dykeman, E.C. A stochastic model for simulating ribosome kinetics *in vivo*. *PLoS Comput. Biol.* **2020**, *16*, e1007618.

[67] Lizzano, R.A.; Yang, B.; Clippinger, A.J.; Bouchard, M.J. The C-terminal region of the hepatitis B virus X protein is essential for its stability and function. *Virus Res.* **2011**, *155*, 231–239.

[68] Kim, J.H.; Kang, S.; Kim, J.; Ahn, B.Y. Hepatitis B virus core protein stimulates the proteasome-mediated degradation of viral X protein. *J. Virol.* **2003**, *77*, 7166–7173.

[69] Hou, L.; Zhao, J.; Gao, S.; Ji, T.; Song, T.; Li, Y.; Wang, J.; Geng, C.; Long, M.; Chen, J.; et al.. Restriction of hepatitis B virus replication by c-Abl–induced proteasomal degradation of the viral polymerase. *Sci. Adv.* **2019**, *5*, eaau7130.

[70] Cook, D.L.; Gerber, A.N.; Tapscott, S.J. Modeling stochastic gene expression: implications for haploinsufficiency. *Proc. Natl. Acad. Sci. USA* **1998**, *95*, 15641–15646.

[71] Ehlers, I.; Horke, S.; Reumann, K.; Rang, A.; Grosse, F.; Will, H.; Heise, T. Functional characterization of the interaction between human La and hepatitis B virus RNA. *J. Biol. Chem.* **2004**, *279*, 43437–43447.

[72] Hao, R.; He, J.; Liu, X.; Gao, G.; Liu, D.; Cui, L.; Yu, G.; Yu, W.; Chen, Y.; Guo, D. Inhibition of hepatitis B virus gene expression and replication by hepatocyte nuclear factor 6. *J. Virol.* **2015**, *89*, 4345–4355.

[73] Aly, H.H.; Suzuki, J.; Watashi, K.; Chayama, K.; Hoshino, S.i.; Hijikata, M.; Kato, T.; Wakita, T. RNA exosome complex regulates stability of the hepatitis B virus X-mRNA transcript in a non-stop-mediated (NSD) RNA quality control mechanism. *J. Biol. Chem.* **2016**, *291*, 15958–15974.

[74] Murray, J.M.; Wieland, S.F.; Purcell, R.H.; Chisari, F.V. Dynamics of hepatitis B virus clearance in chimpanzees. *Proc. Natl. Acad. Sci. USA* **2005**, *102*, 17780–17785.

[75] Murray, J.M.; Purcell, R.H.; Wieland, S.F. The half-life of hepatitis B virions. *Hepatology* **2006**, *44*, 1117–1121.

[76] Guo, J.T.; Pryce, M.; Wang, X.; Barrasa, M.I.; Hu, J.; Seeger, C. Conditional replication of duck hepatitis B virus in hepatoma cells. *J. Virol.* **2003**, *77*, 1885–1893.

[77] Xu, C.; Guo, H.; Pan, X.B.; Mao, R.; Yu, W.; Xu, X.; Wei, L.; Chang, J.; Block, T.M.; Guo, J.T. Interferons accelerate decay of replication-competent nucleocapsids of hepatitis B virus. *J. Virol.* **2010**, *84*, 9332–9340.

[78] Ciupe, S.M.; Ribeiro, R.M.; Nelson, P.W.; Dusheiko, G.; Perelson, A.S. The role of cells refractory to productive infection in acute hepatitis B viral dynamics. *Proc. Natl. Acad. Sci. USA* **2007**, *104*, 5050–5055.

[79] Aunins, T.R.; Marsh, K.A.; Subramanya, G.; Uprichard, S.L.; Perelson, A.S.; Chatterjee, A. Intracellular hepatitis C virus modeling predicts infection dynamics and viral protein mechanisms. *J. Virol.* **2018**, *92*, e02098–17.

[80] Jaks, E.; Gavutis, M.; Uzé, G.; Martal, J.; Piehler, J. Differential receptor subunit affinities of type I interferons govern differential signal activation. *J. Mol. Biol.* **2007**, *366*, 525–539.

[81] De Weerd, N.A.; Samarajiwa, S.A.; Hertzog, P.J. Type I interferon receptors: biochemistry and biological functions. *J. Biol. Chem.* **2007**, *282*, 20053–20057.

[82] Roe, S.M.; Prodromou, C.; O'Brien, R.; Ladbury, J.E.; Piper, P.W.; Pearl, L.H. Structural basis for inhibition of the Hsp90 molecular chaperone by the antitumor antibiotics radicicol and geldanamycin. *J. Med. Chem.* **1999**, *42*, 260–266.

[83] Fung, J.; Lai, C.L.; Seto, W.K.; Yuen, M.F. Nucleoside/nucleotide analogues in the treatment of chronic hepatitis B. *J. Antimicrob. Chemother.* **2011**, *66*, 2715–2725.

[84] van Hemert, F.J.; Berkhout, B.; Zaaijer, H.L. Differential binding of tenofovir and adefovir to reverse transcriptase of hepatitis B virus. *PLoS One* **2014**, *9*, e106324.

[85] Dykeman, E.C.; Stockley, P.G.; Twarock, R. Building a viral capsid in the presence of genomic RNA. *Phys. Rev. E* **2013**, *87*, 022717.

[86] Bartlett, D.W.; Davis, M.E. Insights into the kinetics of siRNA-mediated gene silencing from live-cell and live-animal bioluminescent imaging. *Nucleic Acids Res.* **2006**, *34*, 322–333.

[87] Carey, J.; Uhlenbeck, O.C. Kinetic and thermodynamic characterization of the R17 coat protein-ribonucleic acid interaction. *Biochemistry* **1983**, *22*, 2610–2615.
